# Topography and functional traits shape the distribution of key shrub plant functional types in low-Arctic tundra

**DOI:** 10.3389/fpls.2025.1724838

**Published:** 2026-01-07

**Authors:** Daryl Yang, Wouter Hantson, Kenneth J. Davidson, Julien Lamour, Bailey D. Morrison, Verity G. Salmon, Tianqi Zhang, Kim S. Ely, Charles E. Miller, Daniel J. Hayes, Stephen Baines, Alistair Rogers, Shawn P. Serbin

**Affiliations:** 1 Environmental Sciences Division, Oak Ridge National Laboratory, Oak Ridge, TN, United States; 2 Environmental and Climate Sciences Department, Brookhaven National Laboratory, Upton, NY, United States; 3 Department of Ecology and Evolution, Stony Brook University, Stony Brook, NY, United States; 4 School of Forest Resources, University of Maine, Orono, ME, United States; 5 WSL Institute for Snow and Avalanche Research SLF, Alpine Environment and Natural Hazards, Davos, Dorf, Switzerland; 6 American Forests, Washington, WA, United States; 7 Centre de Recherche sur la Biodiversité et l’Environnement (CRBE), Université de Toulouse, CNRS, IRD, Toulouse INP, Université Toulouse 3 – Paul Sabatier (UT3), Toulouse, France; 8 Department of Civil and Environmental Engineering, University of California, Merced, Merced, CA, United States; 9 Climate and Ecosystem Sciences Division, Berkeley National Laboratory, Berkeley, CA, United States; 10 Jet Propulsion Laboratory, California Institute of Technology, Pasadena, CA, United States; 11 Biospheric Sciences Laboratory (Code 618), NASA Goddard Space Flight Center, Greenbelt, MD, United States

**Keywords:** arctic, shrubification, patch dynamics, environmental limits, alder, willow

## Abstract

The expansion of shrubs in the Arctic tundra fundamentally modifies land-atmosphere interactions. However, it remains unclear how shrub distribution and expansion differ across key species due to challenges with discriminating tundra plant species at regional scales. Here, we combined multi-scale, multi-platform remote sensing and *in situ* trait measurements to elucidate the distribution patterns and primary controls of two representative deciduous-tall-shrub (DTS) genera, *Alnus* and *Salix*, in low-Arctic tundra. We show that topographic features were a key control on DTSs, creating heterogeneous, but predictable distributions of *Alnus* and *Salix* fractional cover (fCover). *Alnus* was more tolerant of elevation and slope and was found on hilly uplands (slope >10°) within a specific elevational band (200–400 m above sea level [MSL]). In contrast, *Salix* occurred at lower elevations (50–300 m MSL) on gentler slopes (3-10°) and required adequate soil moisture associated with its profligate water use. We also show that niche differentiation between *Alnus* and *Salix* changed with patch size, where larger patches were more specialized in resource requirements than individual plants of *Alnus* and *Salix*. To understand what constrains the growth of DTSs at locations with low fCover, we developed environmental limiting factor models, which showed that topography limits the upper bound of *Alnus* and *Salix* fCover in 69.2% and 48.7% of the landscape, respectively. These findings highlight a critical need to better understand and represent topography-controlled processes and functional traits in regulating shrub distribution, as well as a need for more detailed species classification to predict shrubification in the Arctic.

## 1 Introduction

Northern high-latitude ecosystems are warming faster than any other region on Earth, driving associated changes in surface temperatures, precipitation, albedo, and sea ice feedbacks (Chapin et al., 2005; Hinzman et al., 2013; Jenkins and Dai, 2021). Ecosystems are responding to this warming in ways that may accelerate or slow down climate change. In particular, changes in vegetation composition influence processes that control the thaw of permafrost, which stores up to 50% of the world’s below-ground carbon (Sistla et al., 2013; Schuur et al., 2015). Over the past 40 years, increased shrub cover and height (known as “Arctic shrubification”) have been observed in many areas, including the Canadian Arctic (Lantz et al., 2010; Myers-Smith et al., 2011b; Tremblay et al., 2012), Arctic Russia (Forbes et al., 2010), and northern Alaska (Sturm et al., 2001b; Tape et al., 2006). This increase in shrub dominance is expected to have far-reaching impacts on soil-plant-atmosphere interactions, altering the energy balance and carbon budget of these ecoregions (DeMarco et al., 2014; Vowles and Björk, 2019; Mekonnen et al., 2021a) and contributing to an overall greening trend of the Arctic (Bennett et al., 2022; Frost et al., 2023).

Tundra ecosystems are known to be typically covered by short-stature plants (typically<0.4 m, such as moss, lichen, graminoids, forbs, and dwarf to low shrubs), and landscapes in this region are often a complex mosaic of vascular and non-vascular vegetation, thaw ponds, drainages, seasonal snow, and exposed rocks. The establishment of deciduous tall shrubs (DTSs), especially those that can grow more than 2 m tall (Walker, 2000; Breen et al., 2020), can substantially change landscape structure, with multifaceted direct or indirect impacts on ecosystem processes (Myers-Smith et al., 2011a; Mekonnen et al., 2021a). For example, compared to short-stature tundra plants, DTSs have a much larger leaf area and woody biomass that can enhance ecosystem-scale uptake of atmospheric CO_2_, creating a negative feedback on climate warming (Shaver and Chapin, 1991; Cahoon et al., 2012). The larger and more extensive root systems of DTSs also enlarge the plant–soil interface (Iversen et al., 2015), which, together with greater litter inputs, have been associated with positive rhizosphere priming that increases carbon release in the Arctic (Friggens et al., 2025). On the other hand, the taller and generally darker canopies of DTSs reduce surface albedo, increasing solar radiation absorption during the snow-free season as well as during the winter when woody material extends above the snowpack (Blok et al., 2011; Lawrence and Swenson, 2011; Zhang et al., 2018). Tall shrub patches also act as “snow fences”, increasing localized snow accumulation and resulting in warmer winter soil temperatures and faster litter decomposition (Sturm et al., 2001a; DeMarco et al., 2014; Paradis et al., 2016; Kropp et al., 2020; Bennett et al., 2022) while also altering micro-scale hydrology (Bring et al., 2016). The formation of large, closed DTS canopies also affects community assembly, with a shuffling of strategies related to resource allocation (Parker et al., 2021), water and nutrient cycling (Salmon et al., 2019; Devotta et al., 2021), and impacts plant and animal biodiversity (Joly et al., 2007; Wheeler et al., 2018).

Despite the important impacts of DTSs on landscapes and ecosystems, the processes and mechanisms that control DTS distribution remain poorly understood, leading to significant uncertainties in predicting the fate of the Arctic under future climate scenarios (Martin et al., 2017). To date, increases in shrub cover and height have been linked mainly to warming summer conditions and permafrost thaw (Fraser et al., 2011; Andreu-Hayles et al., 2020; Chen et al., 2021). However, tundra landscapes contain a commensurate degree of spatial heterogeneity in DTS distribution and non-uniform response to climate change (Berner et al., 2020) that is potentially associated with fine-scale variability in climate, topography, and soil conditions, which remains poorly studied. In addition to fine-scale environmental variability, several tradeoffs may also contribute to the emergent spatial heterogeneity in DTSs. Particularly, while warming temperatures and permafrost thaw provide improved vegetation growth conditions across the Arctic (Andreu-Hayles et al., 2020), many site factors (e.g., topographic control, water, and nutrient availability) may remain unfavorable for DTS establishment given the generally harsh environments in this region (Tape et al., 2012; Myers-Smith et al., 2015; Chen et al., 2021). The presence of these limiting environmental factors could constrain or outweigh warming-driven enhancements to DTS expansion. For example, multiple studies have shown that shrub expansion response to climate change is highly moisture-dependent (Martin et al., 2017; Ackerman et al., 2017), with wet sites showing a higher sensitivity (i.e., more expansion) to climate than dry sites (Naito and Cairns, 2011; Myers-Smith et al., 2015). Such variability in the controls of DTSs is important to quantify, but has so far been difficult using existing approaches, due to both observational challenges to quantify the spatial heterogeneity in shrub distribution and related fine-scale environmental conditions and methodological challenges to accurately identify the controlling factors from a suite of environmental variables (Greenberg et al., 2015; Swanson, 2015).

DTS species also differ in their spatial distribution and responses to climate change. For example, in northern Alaska and the Canadian Arctic, a high cover and seedling density of willow species (e.g., *Salix pulchra*) have been observed at sites with elevated soil moisture along rivers, streams, and drainages (Schickhoff et al., 2002; Myers-Smith et al., 2011b; Swanson, 2015), while high cover of alder species (e.g., *Alnus viridis*) are commonly found on hillslopes with more summer warmth and lower soil PH (Swanson, 2015; Mekonnen et al., 2021b). This difference in spatial distribution reflects the diverse survival strategies of DTS species and potentially different responses to climate change, which is partly evidenced by a recent study showing thermal segregation in the spatial expansion of *Alnus* and *Salix* in Alaska’s Chugach Mountains (Rinas et al., 2017). However, at present, regional analyses of shrub distribution or expansion—a scale important for capturing the influence and interactions of heterogenous environmental conditions that are hard to fully represent at site scale (e.g., topographic variation and permafrost distribution; Turner, 1989)—generally do not differentiate between species or genera (Tape et al., 2012; Andreu-Hayles et al., 2020). As a result, the patterns and drivers of the different distributions across DTS species, as well as their trajectory under different climate change projections, remain poorly understood.

One of the primary constraints to a species-specific (or genus-specific) understanding of DTSs has been the lack of high-resolution data that are capable of distinguishing between shrub species, as well as capturing fine-scale variations in environmental conditions (e.g., microclimate, topography, and soil properties). In the past four decades, satellite remote sensing has been used to map vegetation types in the Arctic (Stow et al., 2004; Walker et al., 2005; Olthof and Fraser, 2007; Davidson et al., 2016; Macander et al., 2017; Raynolds et al., 2019). However, due to coarse spatial (>30m) and spectral (multi-spectral bands of 40 to 100 nm wide) resolutions, vegetation maps derived from these data typically lump DTS species into a single class (Wang et al., 2019), or sometimes even mixed with other low shrub or tree species (Walker et al., 2005). Climate and soil properties are also commonly provided at a coarse resolution (typically >1 km) that fails to capture the fine-scale environmental gradients that control shrub establishment, growth, and dispersal (Niittynen et al., 2020a, 2020b; Dobbert et al., 2021). On the other hand, ground-based studies can provide detailed information on vegetation and environmental conditions (Niittynen et al., 2020a, 2020b; Aalto et al., 2021), but are limited to small areas, usually focusing on single hillslopes, and require intensive fieldwork that is costly and time-consuming in the Arctic.

The synthesis of newer, multi-scale observations (ground, unoccupied aerial systems [UASs], airborne, and satellite) provides a unique opportunity to address these problems associated with scaling between satellite and ground-based studies (Yang et al., 2022). In recent years, efforts have been made to collect UAS (e.g., Assmann et al., 2020; Yang et al., 2020) and airborne observations (e.g., NASA’s Airborne Visible/Infrared Imaging Spectrometer - Next Generation [AVIRIS-NG] and Land Vegetation and Ice Sensor [LVIS]) in the Arctic region (Miller et al., 2019, 2024; Montesano et al., 2022). These novel observations enhance our ability to map, upscale, and monitor tundra vegetation with a fidelity that was previously impossible (Nelson et al., 2021; Yang et al., 2022). For example, by integrating high-resolution UAS and hyperspectral AVIRIS-NG data, Yang et al. (2023) showed that the fractional cover (fCover) of spectrally similar plant functional types (PFTs), like alder and willow, can be accurately differentiated from AVIRIS-NG imagery. This advance in vegetation monitoring, combined with other ground, remotely-sensed, or model-simulated vegetation (e.g., leaf traits) and environmental (e.g., climate, soil, and topography) data may enable novel research approaches to quantify the controls on vegetation distribution.

**Figure 1:**
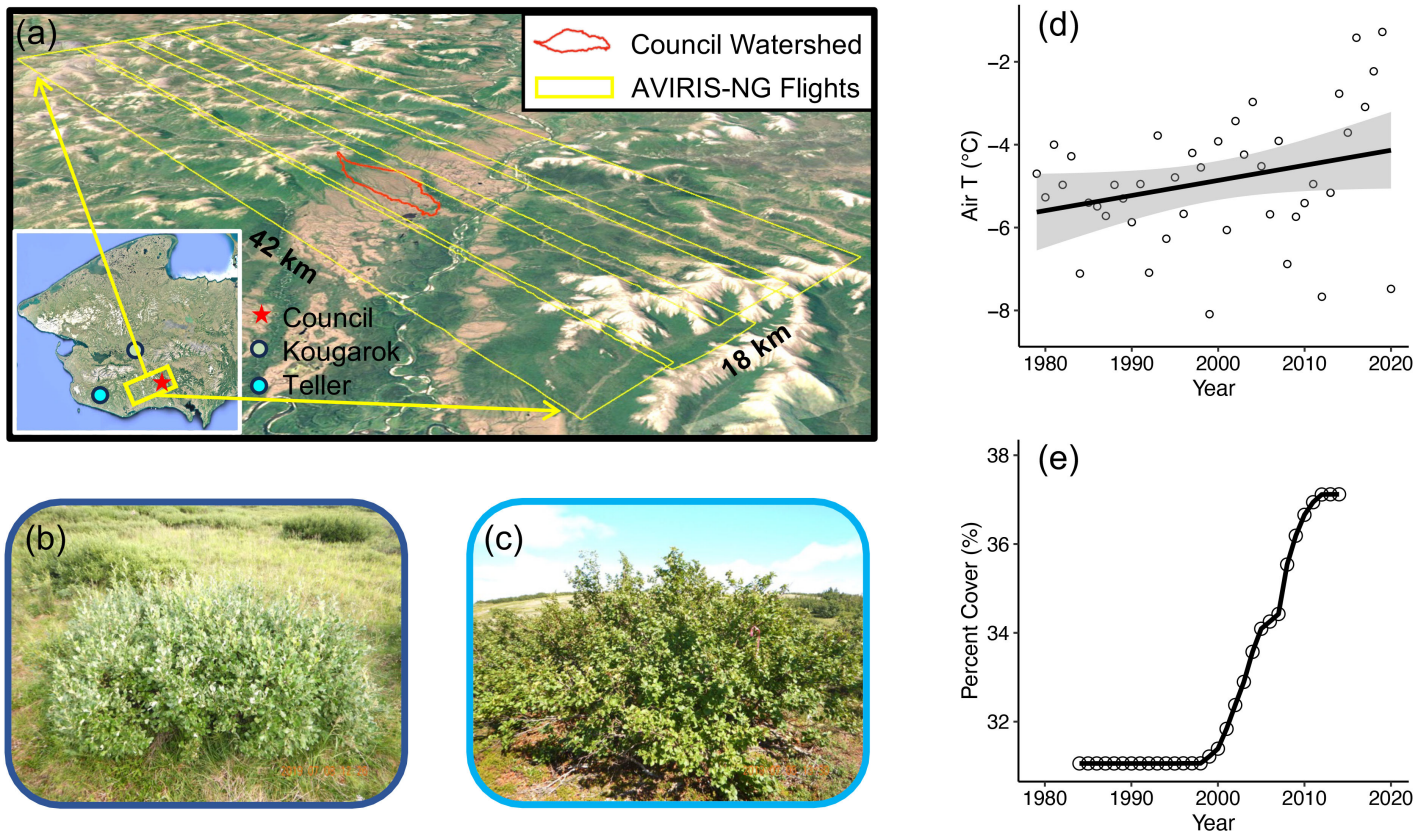
**(a)** Location of the study area on the Seward Peninsula; **(b)** Field photo of a representative *Salix* species (*Salix glauca*) and **(c)** Field photo of a representative *Alnus* species (*Alnus viridis*); **(d)** Historical air temperature change at Council since 1980; **(e)** Total deciduous tall shrub (DTS) cover change within the studied region since 1984. The yellow rectangles in panel **(a)** indicate the four Airborne Visible/Infrared Imaging Spectrometer Next Generation (AVIRIS-NG) flights used for mapping *Alnus* and *Salix* fractional cover (fCover). Air temperature data in **(d)** is derived from ERA5-land climate reanalysis data (https://cds.climate.copernicus.eu/cdsapp#!/dataset/reanalysis-era5-land?tab=overview). The DTS percent cover in **(e)** is calculated from a Landsat-derived land cover product from the Arctic Boreal Vulnerability Experiment (ABoVE) (https://daac.ornl.gov/ABOVE/guides/Annual_Landcover_ABoVE.html).

In this study, we built on these advances in multi-scale, multi-platform remote sensing data and approaches to investigate the distribution and primary controls and limits of two key DTS genera, *Alnus* (**Figure 1c**) and *Salix* (**Figure 1b**), in a low-Arctic tundra landscape in western Alaska. To do this, we mapped pixel-wise fCover for *Alnus* and *Salix* at 5.2 m resolution using a combination of UAS and AVIRIS-NG data. This high-resolution shrub fCover dataset was then combined with climate, topography, and soil data, along with *in situ* leaf trait measurements, to investigate the spatial patterns and primary controls of *Alnus* and *Salix* distribution. Using these data and an environmental limiting factor (ELF) modeling approach, we also determined the spatial distribution of factors that constrain low *Alnus* and *Salix* fCover. With these analyses, we aim to answer three fundamental questions: (1) How does DTS distribution vary across low-Arctic landscapes and what are the primary drivers? (2) To what extent does the spatial distribution differ between *Alnus* and *Salix* and how does it associate with environmental drivers and functional traits? (3) What are the dominant environmental factors that constrain the current spatial distribution of *Alnus* and *Salix* and what is the potential for *Alnus* and *Salix* expansion under these constraints?

## 2 Materials and methods

### 2.1 Study area

Our study focused on a low-Arctic tundra landscape located in the southeast Seward Peninsula (**Figure 1a**), in the vicinity of the village of Council. The Seward Peninsula is located in western Alaska, an area highly exposed to climate and vegetation change (**Figure 1d, e**; Rupp et al., 2000; Lloyd et al., 2002). The study area (**Figure 1a**) was approximately 42 × 18 km, covering a diversity of tundra, shrub, and forest vegetation types (Lloyd et al., 2002; Kim et al., 2014), and included strong environmental and topographic variation (from sea level to 650 m MSL). As one travels inland and upland, the dominant tall shrub species shifts gradually from *Salix* spp. to *Alnus* spp., which is representative of the general *Salix* and *Alnus* distribution in western Alaska (Swanson, 2015). This region of the Seward Peninsula was likely to be last glaciated during the early Pleistocene (Kaufman and Hopkins, 1986; Kaufman and Manley, 2004) and permafrost here is discontinuous (Hinzman et al., 2003; Lloyd et al., 2003). The mean annual temperature (MAT) at Council from 2018 to 2020 was -0.98 °C (Krassovski and Riggs, 2019). The mean annual growing season precipitation measured in 2011 and 2012 was 265 mm (Kim et al., 2014), and the maximum snowpack depth measured in 2001 and 2002 was 115 cm (Sturm, 2005). As part of the US Department of Energy’s effort to evaluate understanding and model representation of low-Arctic ecosystems, the Next-Generation Ecosystem Experiments Arctic (NGEE-Arctic) project (https://ngee-arctic.ornl.gov/) established a watershed study site in this region (red polygon in **Figure 1a**; henceforth called ‘Council’ site).

### 2.2 *Alnus* and *Salix* cover

The cover of *Alnus* and *Salix* across the landscape was mapped using imaging spectroscopy data from AVIRIS-NG collected as part of NASA’s Arctic Boreal Vulnerability Experiment (ABoVE; Miller et al., 2019, 2025). This dataset has a high spectral resolution (~5 nm) and continuous spectral coverage (380 nm - 2510 nm), and can effectively differentiate between *Alnus* and *Salix* (Yang et al., 2023). For this study, we used four parallel flights (**Figure 1a**) collected on July 9th, 2019, between 10:40 and 11:20 local time. The flights were carried out under clear sky conditions at an average flight altitude of ~5.4 km, which provided a ground sampling distance of ~5.2 m. The resulting images were orthorectified and preprocessed to apparent surface reflectance by NASA Jet Propulsion Laboratory (JPL; Thompson et al., 2015; Miller et al., 2024, 2025). We further processed the images to remove topographic and bidirectional reflectance distribution function (BRDF) effects using the modified sun-canopy-sensor topographic method (Soenen et al., 2005) and a quadratic function of the volumetric scattering term of the Ross-Thin BRDF model (Roujean et al., 1992; Lucht et al., 2000).

**Figure 2:**
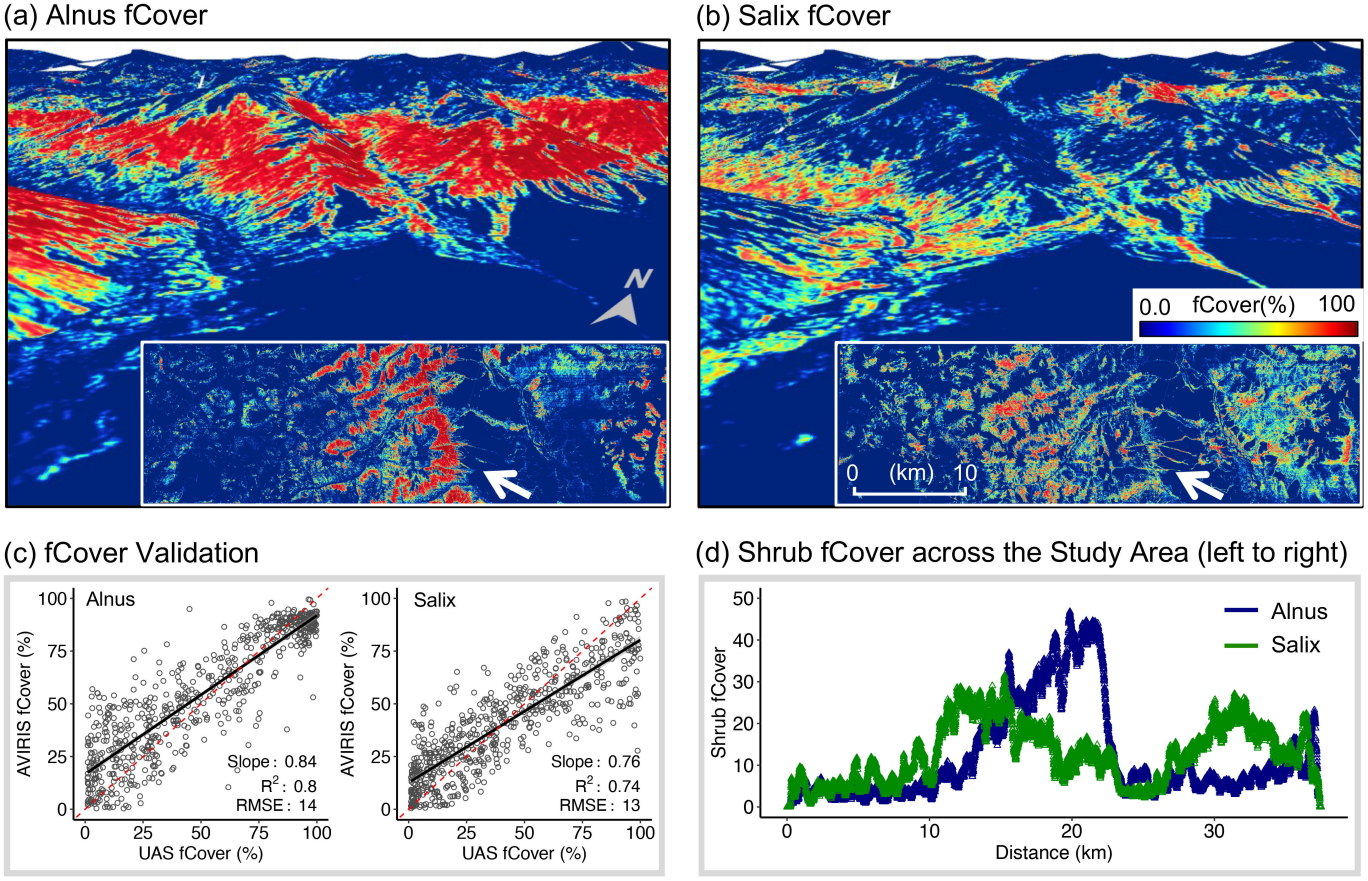
**(a, b)***Alnus* and *Salix* fractional cover (fCover) derived from imaging spectroscopy data collected with AVIRIS-NG; **(c)** Validation of AVIRIS-NG derived shrub fCover against validation data derived from very-high-resolution unoccupied aerial system (UAS) imagery; **(d)** Distribution of average *Alnus* and *Salix* fCover across the AVIRIS-NG surveyed region (from left to right). The white arrows in **(a, b)** indicate the viewing angle for generating the 3-dimensional maps.

To map *Alnus* and *Salix* cover from AVIRIS-NG images, we used the fCover scaling models developed by Yang et al. (2023). Briefly, the scaling models linked ground-truth fCover with AVIRIS-NG spectra using partial least squares regression (PLSR) and predicted pixel-wise fCover for 12 Arctic PFTs, including *Alnus* (mainly *Alnus viridis* spp) and *Salix (*mainly *Salix pulchra, Salix glauca, Salix richardsonni, and Salix alaxensis)* DTSs, with high accuracy (root mean square error [RMSE]<13%). In this study, we applied the model developed by Yang et al. (2023) to each of our four parallel AVIRIS-NG flights and produced a mosaicked fCover map (**Figure 2**) that covered the study area. We assessed the uncertainty (**Supplementary Figure S1**) of the predicted fCover using an ensemble of 500 PLSR models and the final PFT fCover was calculated as the mean of the 500 models Yang and Serbin (2024a).

We independently validated the PLSR-predicted fCover using very-high-resolution (2 cm) UAS classification maps collected at four locations along the Council Road (**Supplementary Figure S2**). These maps were created using a combination of UAS-derived red-green-blue (RGB) and canopy height model (CHM) and an object-based random forest classification in Python (version 3.9). The maps had an overall accuracy >88%, including *Alnus* and *Salix* classification. See **Supplementary Table S1** and Yang and Serbin (2024b) for details about this dataset and accuracy assessment. For this study, we aggregated the UAS maps to produce *Alnus* and *Salix* fCover at 5.2 m resolution and compared them against AVIRIS-NG derived fCover. Taking UAS-derived fCover as ground truth, we conducted linear regression against AVIRIS-NG derived fCover, and calculated slope, coefficient of determination (R^2^), and root mean square error (RMSE). The result showed that AVIRIS-NG derived fCover were estimated with R^2^ >0.74, and RMSEs<14% (**Figure 2c**), similar to accuracy reported in Yang et al. (2023). The regression slope is >0.76 for both *Alnus* and *Salix*, which, combined with the high R^2^ and low RMSEs, indicates good agreement between AVIRIS-NG and UAS-derived fCover.

### 2.3 Environmental data

We investigated 13 environmental variables as potential controls of *Alnus* and *Salix* distribution, which can be primarily grouped into three categories: climate, topography, and soil properties (**Table 1** & **Supplementary Figure S3**). For climate, eight variables that were representative of energy and water resource distribution were used, including radiation, potential evapotranspiration (ET0), actual evapotranspiration (AET), water deficit (ET0 - AET), rain, snow, annual maximum temperature (T_max_), and annual minimum temperature (T_min_). To account for the high spatial variation in micro-climate, we used down-scaled, high-resolution (60 m) climate data representing contemporary conditions (1975 - 2005) for the study area (Morrison and Greenberg, 2019).

**Table 1:** Environmental drivers used in this study for examining the controls of *Alnus* and *Salix* distribution.

Data type	Time period	Resolution	Included variables	Unit
Climate	1975 - 2005	60 m	Potential Evapotranspiration (ET0)	mm
			Apparent Evapotranspiration (AET)	mm
			Water Deficit (ET0-AET)	mm
			Radiation	w/m^2^
			Precipitation	mm
			Snow Water Equivalent	mm
			Maximum Temperature (Tmax)	K
			Minimum Temperature (Tmin)	K
Topography	NA	32 m	Elevation	m
			Slope	degree
			Topographic Wetness Index (TWI)	N/A
Soil	1975 - 2005	500 m	Active Layer Depth (ALD)	m
			Annual Ground Temperature (AGT)	K

Topography data of the study area was sourced from ArcticDEM 32 m resolution digital elevation model (DEM) (https://www.pgc.umn.edu/data/arcticdem/). From the DEM, we derived slope and topographic wetness index (TWI) using the System for Automated Geoscientific Analyses (SAGA) module in QGIS. Here, TWI is calculated as a function of slope and upstream contributing area (Equation 1) and is an indicator of soil moisture across topographic gradients (Beven and Kirkby, 1979). Typically, a large TWI value indicates high soil moisture, and vice versa.

(1) $$TWI=ln\left(\frac{SCA}{\text{tan}\left(\varphi \right)}\right)$$

where *SCA* is upstream contributing area and *φ* is slope in radians. We implemented the TOPMODEL embedded in SAGA to calculate TWI (Ambroise et al., 1996; Beven et al., 2021). Here we also included DEM (i.e., elevation), which has been shown important for understanding shrub distribution in the Arctic (Derkacheva et al., 2025; Rinas et al., 2017). We note that DEM is defined as a topographic variable here following existing literature (Amatulli et al., 2018; Jucker et al., 2018), but we interpret it as a combination of topography and local climate in this study.

Though fine-scale permafrost properties (e.g., active layer depth [ALD] and annual ground temperature [AGT]) are potentially important factors determining tundra vegetation distribution (Grünberg et al., 2020; Niittynen et al., 2020b), acquiring high-resolution soil data is challenging in the Arctic. NASA’s Airborne Synthetic Aperture Radar (SAR) has been used to derive ALD at the Council site, but it did not cover our full study area. For this, we turned to a model-simulated ALD and AGT dataset that has a 500 m spatial resolution and was rigorously calibrated and validated using ground measurements across the Seward Peninsula (Debolskiy et al., 2019, 2020). The dataset includes retrospective and predicted ALD and AGT covering the historic and projected conditions from 1901 - 2100, with AGT simulated at 10 soil depths (Debolskiy et al., 2019). For this study, we used shallow-soil AGT within 50 cm depth which has been shown to be strongly linked with Arctic vegetation distribution (Léger et al., 2019; Kropp et al., 2020). The mean ALD and AGT during 1975 - 2005 (to match the climate data) was then computed to represent contemporary belowground conditions. It is noted that this study focused on spatial drivers underlying the distributional patterns of *Alnus* and *Salix* and leveraged the best available high-resolution climate and soil datasets for the region. Though climate change has occurred between 1975–2005 and the period of our field and remote sensing data collection (2016–2023), the impacts of temporal mismatch on our analysis are likely marginal because shrub distribution responses to climate change could lag by several decades (Corlett and Westcott, 2013; Liu et al., 2022).

### 2.4 Statistical analyses

#### 2.4.1 Characterizing spatial patterns of DTS distribution

To investigate the spatial pattern (e.g., patch size) of DTS distribution, we performed a spatial analysis using semi-variograms (a measure of spatial autocorrelation; Garrigues et al., 2006) on the 5.2 m resolution AVIRIS-NG-derived *Alnus* and *Salix* fCover. To reduce the computational cost, we randomly sampled 5% of the ca. 34 million pixels. The samples were then fitted with a variogram model using the *gstat* package in *R* (version 4.1.0; R Core Team, 2021). Here, we drew samples and fitted the variogram models for *Alnus* and *Salix* separately. The spherical model, which was identified as the best model (highest pseudo r-squared score) by *gstat*, was used to fit the variogram for both *Alnus* and *Salix*. To account for model uncertainty, we repeated the random sampling and variogram fitting procedure 20 times and calculated the mean and standard deviation of the 20 variograms.

It is noted that areas dominated by boreal forests (identified using AVIRIS-NG pixels that are dominantly covered by evergreen and deciduous tree PFTs in fCover maps from Yang et al., 2023) were excluded from this variogram analysis, as well as later analyses because DTSs in these areas are confounded by biological interactions with spruce tree species other than environmental controls (Greenwood and Jump, 2014). In addition, as optical remote sensing can only observe the top layer of vegetation, the cover of *Alnus* and *Salix* (often overshadowed by taller spruces) could be underestimated in forested regions (Kristensen et al., 2015).

#### 2.4.2 Determining drivers of spatial variation in DTS cover

We explored the drivers of the spatial variation in *Alnus* and *Salix* fCover using a random forest (RF) modeling approach to determine the importance of each environmental driver variable for explaining the variation in fCover. To do this, we first determined the best scale for establishing associations between DTS fCover and environmental drivers. This is because while DTS cover could possess high variability at very-fine scales (e.g,<5 m), this variability may not be explained by landscape-scale environmental gradients but by other local site factors, such as species competition, dispersal limitations, and cryogenic soil processes, that are not captured by the environmental data (Frost et al., 2013; Aalto et al., 2021). On the other hand, at coarse scales (e.g., >1000 m), fine-scale spatial variability in DTS cover and the environment are averaged out, resulting in a weaker correlation between the two (Gamon et al., 2020). To determine the best scale to capture shrub-environment interactions, we constructed a RF regression (no. of trees = 500) between DTS fCover and the environmental drivers (climate, topography, and soil; **Table 1**) at a series of pixel resolutions (5, 15, 25, …, 1000 m) and calculated the variance in fCover that is explained by environmental variables at each resolution. In this manner, we determined that 250 m was the best scale (i.e. the resolution at which the variance in fCover was maximally explained; see **Supplementary Figure S4**), and therefore we aggregated all our datasets to 250 m for exploring the drivers of DTS fCover as well as for the remaining analyses.

We assessed the importance of each variable for explaining the spatial variation in fCover using the variable importance for projection (VIP) metric (the percent increase in model mean square error [%InMSE] when a predictor is excluded; Liaw and Wiener, 2002) derived from RF. To account for model uncertainties, we used a bootstrap technique and constructed 100 RF models (Equation 2) at 250 m scale by randomly selecting 75% of the image pixels to train each RF model:

(2) $$fCover_{i}=f\left(Climate_{i}+Topo_{i}+Soil_{i}\right)$$

where *fCover_i_* is the cover of *Alnus* or *Salix* in pixel *i*, and *Climate_i_*, *Topo_i_*, and *Soil_i_* are the climate, topography, and soil variables of pixel *i*. The mean VIP of different predictor variables of the 100 RF models was then computed to determine the primary drivers of shrub fCover. To quantify the differences between *Alnus* and *Salix*, we constructed RF models and calculated mean VIP for them separately. From the RF models, we also produced partial dependence plots (PDPs) that describe how shrub fCover changes over the range of each predictor while keeping other predictors at their mean value (Greenwell et al., 2018). A higher value in PDP indicates a higher sensitivity of shrub fCover to the predictor and vice versa. Similar to VIP, we computed the mean and standard deviation of the 100 RF models to generate a robust estimate of PDPs. These analyses were conducted using tools from R packages *‘randomForest’* (Liaw and Wiener, 2002) and ‘*pdp*’ (Greenwell, 2017).

It is noted that this analysis focused on exploring the primary drivers of fCover distribution and comparison between *Alnus* and *Salix*. We conducted a variable inflation factor (VIF) analysis which showed that multicollinearity among variables did not change the distribution and rank of VIP from our RFs (**Supplementary Figure S5**), similar to what was found in Chavent et al. (2021). For that, we included all 13 predictor variables in our RF models. This ensured that the same model structure was used for both *Alnus* and *Salix* to make consistent comparisons of VIP and PDP.

#### 2.4.3 Quantifying niche differentiation between *Alnus* and *Salix*

In preliminary analysis, we observed that the two genera have an overall west-to-east transition from *Salix* to *Alnus* across the study domain but coexist at various locations (**Supplementary Figure S6**), similar to distribution patterns across the broader Seward Peninsula (Jorgenson et al., 2009). To understand the general patterns and determinants of their coexistence/separation, we conducted two analyses to examine the environmental niche (i.e., fundamental niche) differentiation between the two genera. First, we performed a principal component analysis (PCA) on the combination of climate, topography, and soil data to visualize and inspect the niche space of *Alnus* and *Salix*. Here, considering the potential effects of community size on the differentiation between *Alnus* and *Salix*, we constructed the PCA at a series of fCover ranges (fCover >10%, >20%, …, >60%, >70%). To this end, the climate, topography, and soil data of pixels with shrub fCover >10%, >20%, …, >60%, and >70% were extracted for *Alnus* or *Salix*, respectively, based on the 250 m fCover map. The PCA at each fCover range was then conducted using *‘prcomp’* in R (‘stats’ package; R Core Team, 2021). The percent variance explained by each principal component (PC) was calculated, and the loadings of different environmental variables for each PC were also derived to indicate their contribution to the PCs.

To further quantify the niche differentiation between *Alnus* and *Salix*, we also computed the Euclidean distance between their geometric centers in the hypervolume environmental space (Blonder, 2018), using the *‘hypervolume’* package (version 3.1.0; R Core Team, 2021). Here, all of the 13 climate, topography, and soil variables were included to construct the hypervolume space. Similarly, we computed the Euclidean distance at different ranges of fCover (fCover >0%, 2%, 4%, …, 68%, 70%) to investigate the association between community size and the niche differentiation between *Alnus* and *Salix*.

### 2.5 Exploring possible biological drivers of niche differentiation between *Alnus* and *Salix*

We collected leaf biochemical, structural, and physiological traits to help discern the potential biological drivers of the different distributions between *Alnus* and *Salix*. Given our study domain covered an area (42 × 18 km) that is difficult to access on foot and prohibitively large to sample adequately, we collected and used leaf samples and gas exchange measurements from the nearby NGEE-Arctic field sites at Kougarok and Teller (**Figure 1a**) that are relatively accessible and are respectively representative of *Alnus* and *Salix* distributions in the area (see Salmon et al., 2019; Léger et al., 2019; Yang et al., 2021 & 2023; Schore et al., 2023). In doing so, we assumed that trait differences between *Alnus* and *Salix* are more determined by phenotype and evolutionary history than local climate, and the different distributions between *Alnus* and *Salix* is a result of environmental filtering on traits (Reich et al., 2003; Lin et al., 2015). For details about our trait measurements, see **Table 2** and *Supplemental Material*. We derived six leaf traits (**Table 2**): leaf mass per area (LMA), leaf nitrogen content (LNC), leaf carbon to nitrogen ratio (C: N), maximum carboxylation rate scaled to 25 °C (*V*_cmax.25_), maximum potential electron transport rate scaled to 25 °C (*J*_max,25_), and stomatal slope (*g*_1_). Here, *V*_cmax,25_ and *J*_max,25_ were estimated from A-C_i_ curves using the Farquhar-von Caemmerer-Berry (FvCB) model (Farquhar et al., 1980) and were scaled to the reference temperature 25 °C following Rogers et al. (2017). *g*_1_ was derived from stomatal response curves (Davidson and Serbin, 2024) as modeled using the Unified Stomatal Optimization (USO) model (Equation 3; Medlyn et al., 2011). *g*_1_ is inversely proportional to water use efficiency (WUE) as defined by Cowan and Farquhar (1977).

**Table 2:** Leaf trait measurements of *Alnus* and *Salix* analyzed in this study collected at Kougarok and Teller sites.

Category	Leaf trait	No. of measurements		Years of data collection	Data source
		*Alnus*	*Salix*		
Leaf structure	Leaf mass per area (LMA)	130	202	2016, 2017, 2018, 2019	Rogers et al. (2019); Serbin et al. (2023a, 2023b, 2023c)
Leaf biochemistry	Leaf nitrogen content (LNC)	130	202	2016, 2017, 2018, 2019	Rogers et al. (2019); Serbin et al. (2023a, 2023b, 2023c)
	Leaf carbon nitrogen ratio (C: N)	130	202	2016, 2017, 2018, 2019	Rogers et al. (2019); Serbin et al. (2023a, 2023b, 2023c)
Photosynthesis	Maximum carboxylation rate (*V_cmax_*) at 25 °C	30	13	2019	Rogers et al. (2024); Ely et al. (2024)
	Maximum electron transport rate (*J_max_*) at 25 °C	30	13	2019	Rogers et al. (2024); Ely et al. (2024)
Water use	Stomatal conductance slope (*g_1_*)	15	63	2022, 2023	Davidson and Serbin (2024); Ely et al. (2024)

*Alnus* only occurred at Kougarok sites, but *Salix* occurred at both Kougarok and Teller. For that, trait samples for *Alnus* were only collected at Kougarok site, while traits for Salix were collected at both sites.

(3) $$g_{s}=g_{0\;}+1.6\left(1+\frac{g_{1}}{\sqrt{VPD_{s}}}\right)\frac{A_{n}}{CO2_{s}}$$

where *g_s_* is the stomatal conductance to water vapor (mol m^−2^ s^−1^.), *A_n_* is the net assimilation rate (µmol m^−2^ s^−1^, *g_0_* is the expected *g_s_* when *A_n_*= 0 (here assumed to be 0), VPD*_s_* is the leaf-to-air vapor pressure deficit (kPa), *CO2_s_* is the concentration of CO_2_ at the leaf surface (mol mol^−1^) and *g_1_* is the stomatal slope parameter (kPa^0.5^). Using data shown in **Table 2**, we calculated the mean and standard deviation in LMA, LNC, C: N, *V*_cmax,25_, *J*_max,25_, and *g*_1_ for both *Alnus* and *Salix*. Unpaired t-tests were conducted to examine the significance of difference in each trait between *Alnus* and *Salix*. Along with *g*_1_ measurements, we also collected root-zone soil moisture content using a HydroSense 2 handheld soil moisture sensor (Campbell Scientific, Utah, USA), which was later used to investigate *g*_1_ variation across soil moisture gradients and dominant *Alnus* and *Salix* community types (Alder shrubland, Willow shrubland, Alder-willow-birch shrubland, and Willow birch shrubland; Breen et al., 2020).

### 2.6 Environmental limiting factor modeling for determining DTS expansion potential and ELFs

**Figure 3:**
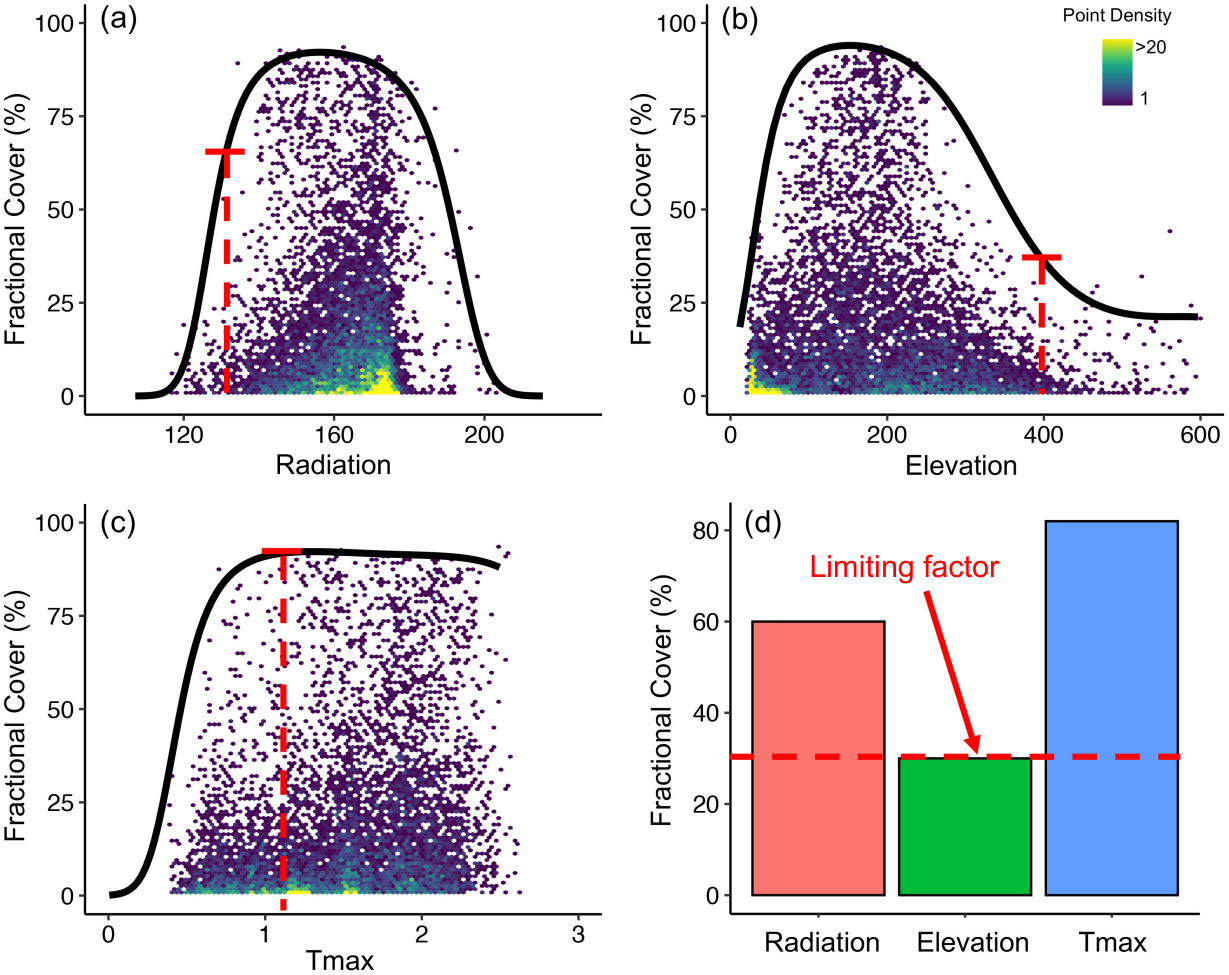
Illustration of the environmental limiting factor modeling (ELF) approach. Here we demonstrated the approach with three variables (radiation, elevation, and Tmax). The black curve in each subplot represents the maximum response of shrub cover to the examined environment driver. The limiting factor at a given location with a specific combination of environment conditions (red dashed lines) is determined as the environmental variable that rendered the minimum shrub cover at the location (bottom right panel). The number of observations in each hex bin is 20.

The cover of DTSs continues to increase with rapid climate warming in the Arctic, but the potential of this increase may vary significantly across space and is ultimately limited by site factors that do not favor shrub growth (Swanson, 2015). Identifying such limiting factors is important to predict DTS change, but has been methodologically challenging. Here, we modeled the potential of DTS expansion in the landscape and corresponding factors that limit this potential, particularly in regions that currently have low DTS cover, using an environmental limiting factor (ELF) modeling approach (Cade and Guo, 2000; Greenberg et al., 2009, 2015). To do this, we assumed that one, and only one, factor (i.e., ELF) controls the maximum shrub fCover at a given time and location (Greenberg et al., 2015). We used two steps to determine the maximum fCover and corresponding ELF (**Figure 3**): (1) we derived per-variable limiting factor models that predict the maximum response (i.e., tolerance) of shrub fCover to each environmental driver (illustrated with three drivers in **Figures 3a–c**); (2) we identified the ELF that yields the minimum potential shrub fCover (**Figure 3d**), i.e., the resource that is the most restricted for shrub growth (Greenberg et al., 2009, 2015; Swanson, 2015).

To construct the per-variable limiting factor models, we used a quantile regression with b-spline from the ‘*quantreg*’ package in R (R Core Team, 2021) to approximate the 99% quantile (black curves in **Figures 3a–c**) of shrub fCover in relation to each of the climate, topography, and soil variables, following (Morrison, 2018). The b-spline was employed for its high flexibility to fit non-linear, curvilinear relationships that commonly exist in ecological data (D’Amario et al., 2019). We fitted the quantile regression at the 250 m optimal scale determined in *Section 2.4* for *Alnus* and *Salix* which yielded 13 per-variable limiting factor models for each genus. To validate the models, we binned each environmental variable into 20 equal-sized intervals and extracted the bin-wise maximum *Alnus* and *Salix* fCover from AVIRIS-NG derived fCover maps (250 m). This dataset was then used as ‘truthing’ to validate the averaged maximum *Alnus* and *Salix* fCover of each bin predicted from the per-variable limiting factor models (Morrison, 2018).

To determine ELFs across the study domain, we applied the per-variable limiting factor models to the 250 m environmental data and produced potential maximum fCover maps for *Alnus* and *Salix* under each of the climate, topography, and soil variables. The ELF of *Alnus* and *Salix* in each pixel was then determined as the variable that produced the minimum potential fCover (as illustrated in **Figure 3d**). To diagnose the differences among climate, topography, and soil properties for limiting shrub growth, we mapped the ELF and potential shrub fCover under each of the three types of environmental variables, respectively, as well as under the combination of all these variables. The growth potential in *Alnus* and *Salix* fCover was then calculated by subtracting the AVIRIS-NG mapped fCover from that predicted with the combination of all environmental variables.

## 3 Results

### 3.1 Spatial patterns of DTS distribution

The fCover maps captured high spatial variability as well as considerable spatial dissimilarity in *Alnus* and *Salix* distributions across the landscape (**Figure 2**). In general, regions with high *Alnus* fCover were concentrated in the middle part and on the east end of the study region (**Figure 2a**), while areas with high *Salix* fCover were relatively scattered across the landscape (**Figure 2b**). At the same time, we observed a higher congregation of both *Alnus* and *Salix* in the middle part of the landscape, which is characterized by mountainous terrain (**Figure 2d**), with large patches of *Alnus* located on hillslopes and shoulders (**Figure 2a**) and *Salix* on toeslopes and along drainages and rivers (**Figure 2b**).

**Figure 4:**
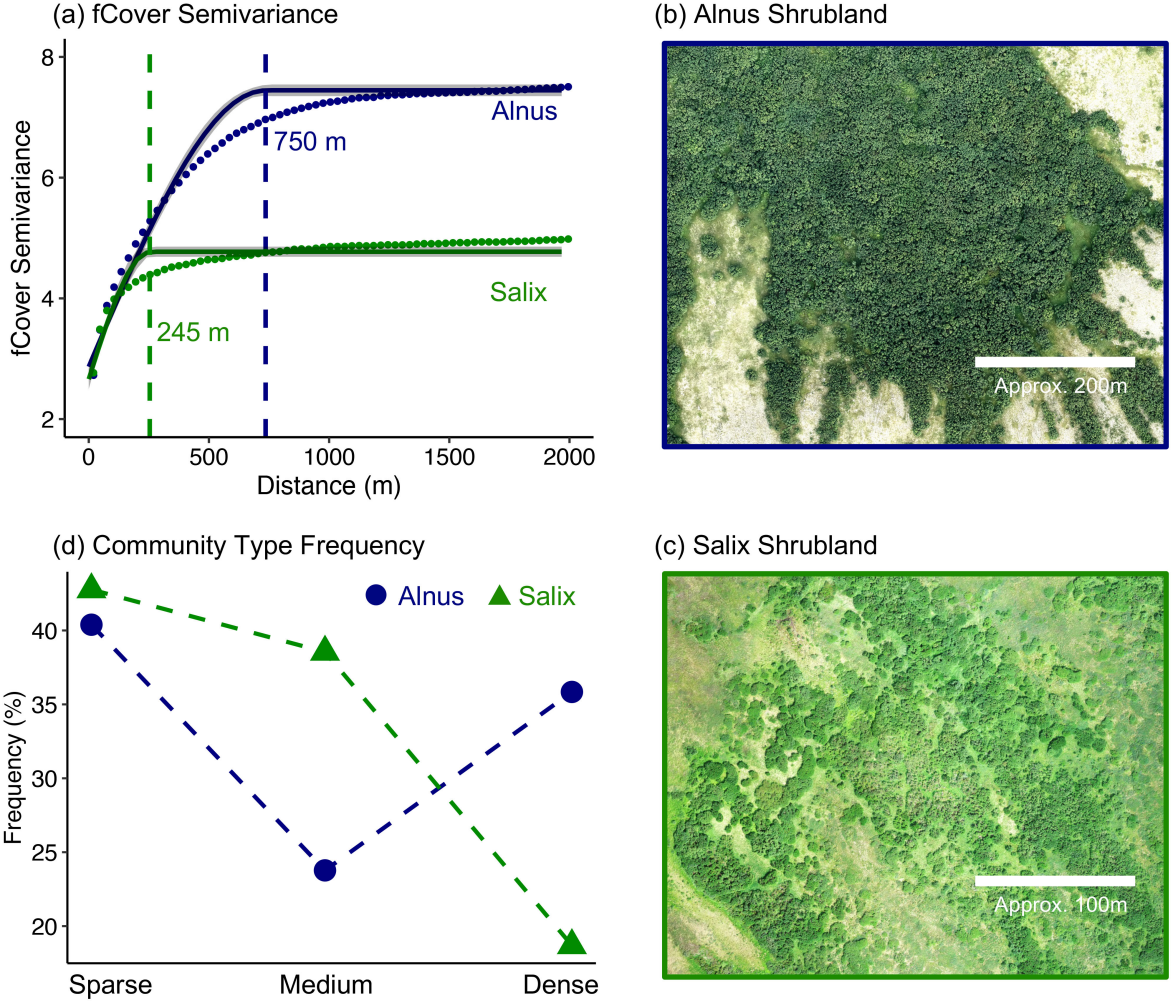
Spatial structure of *Alnus* and *Salix* communities. **(a)** Semivariance of *Alnus* and *Salix* calculated from AVRIS-NG mapped fCover maps (5 m resolution); **(b, c)** Examples of dense *Alnus* and *Salix* communities in tundra landscapes; **(d)** Frequency of plant communities with sparse, medium, and dense Alnus/Salix cover across the studied landscape.

The semi-variogram analysis on the 5.2 m AVIRIS-NG fCover maps showed higher spatial autocorrelation of *Alnus* than that of *Salix* across the landscape (**Figure 4a**). The semivariance of *Alnus* fCover maximized at 750 m, highlighting the potential of *Alnus* to form large, spatially continuous patches (**Figure 4b**). This distance, however, was much smaller for *Salix* (variogram range: 245 m; **Figure 4a**). We considered AVIRIS-NG pixels with shrub fCover of 5 - 25%, 25 - 75%, and >75% as sparse, medium, and high shrub density communities and calculated the frequency of each ‘pixel’ community type within the study area (**Figure 4d**). Aligning with the patterns of spatial autocorrelation, *Alnus* had a much higher frequency of high-density communities than *Salix* (35% for *Alnus*, 19% for *Salix*). Interestingly, we also found that the frequency of sparse-, medium-, and high-density communities showed a ‘V’ shape for *Alnus*, with a much higher frequency of sparse- and high-density communities than medium-density communities (dark blue line in **Figure 4d**). In contrast, the frequency of *Salix* community types decreased with increasing density (green line in **Figure 4d**).

### 3.2 Drivers of spatial variation in DTS cover

**Figure 5:**
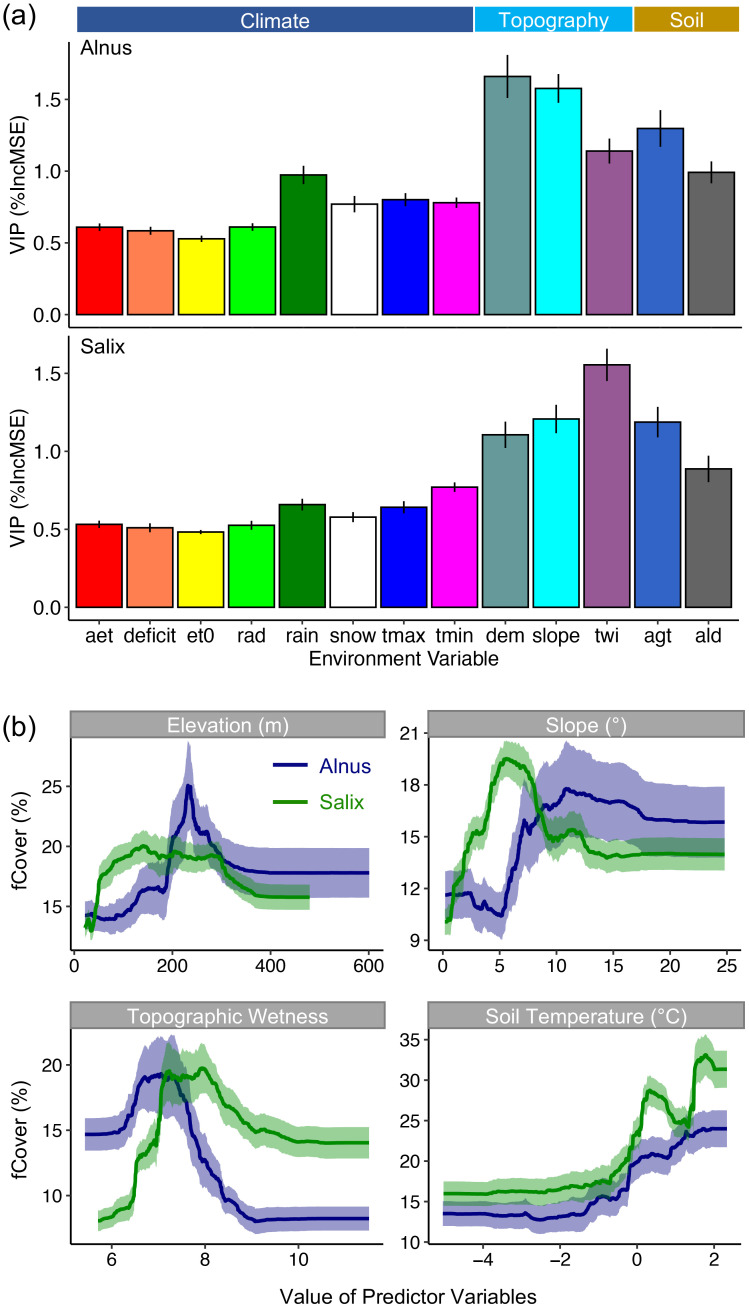
**(a)** Importance of different environmental drivers for describing the spatial variation in *Alnus* and *Salix* fCover derived from random forest (RF) modeling analysis. Here we use the percent increase in model mean square error [%InMSE] as a proxy of variable importance for projection (VIP). The error bar represents the standard deviation of the VIPs derived from 100 RF models. **(b)** Partial dependence plot (PDP) for the first four environmental drivers on the VIP rank. Similarly, the ribbon represents the standard deviation of the 100 RF models.

Among the three types of environmental variables, topographic variables (elevation, slope, and TWI) showed the highest VIPs for explaining the spatial variation in both *Alnus* and *Salix* fCover, followed by soil and permafrost properties (AGT and ALD; **Figure 5a**). Climate variables displayed low relative VIPs despite their considerable variation across the landscape (**Supplementary Figure S3**). However, we did see that rain, snow, T_max_, and T_min_ had slightly higher VIPs than AET, water deficit, ET0, and radiation. We also observed slight differences in VIPs between *Alnus* and *Salix.* For example, the strongest determinant of *Alnus* fCover was elevation (DEM), followed by slope, AGT, and TWI. In contrast, TWI was the most important determinant of *Salix* fCover, followed by slope, AGT, and elevation. Also, precipitation played a stronger role (i.e., higher VIP) in determining *Alnus* fCover, while T_min_ was more important for *Salix* fCover (dark green and pink bars in **Figure 5a**).

The PDP analysis revealed notable differences in the overall response of shrub fCover to different environmental variables, as well as between *Alnus* and *Salix* (**Figure 5b**). In particular, a high *Salix* fCover response was observed at lower elevations (50–300 m), gentler slopes (3 - 10°), and higher topographic wetness (7 - 12), as compared to *Alnus* which had a high fCover response at higher elevations (200–400 m), steeper slopes (10 - 25°), and lower topographic wetness (5 - 7). Interestingly, we observed a peaked response with *Alnus* fCover at an elevation of approximately 200–360 m (top-left panel in **Figure 5b**), indicating a potential concentration of *Alnus* shrubs at this specific elevational band.

### 3.3 Environmental niche differentiation between *Alnus* and *Salix*

**Figure 6:**
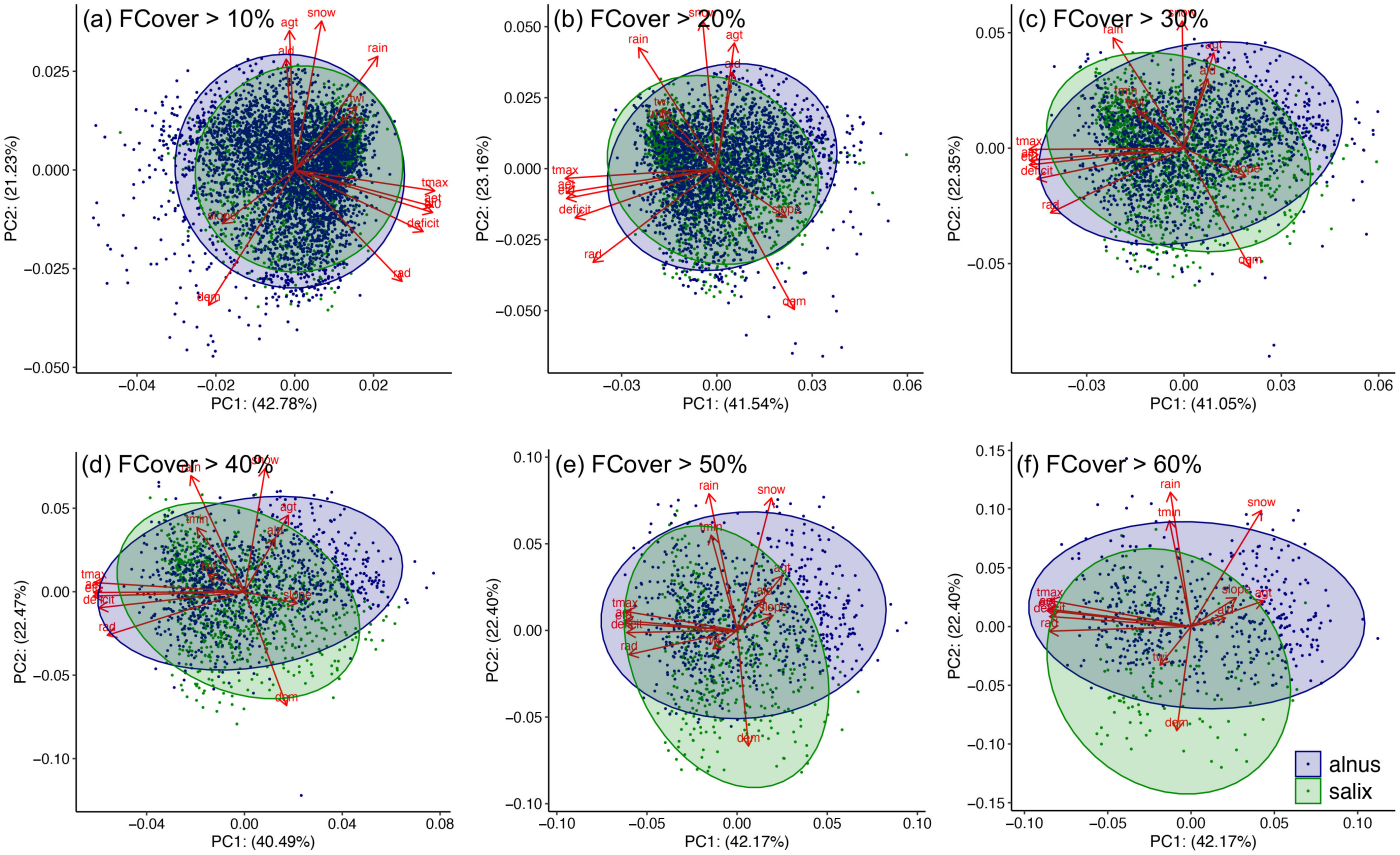
Results of principal component analysis (PCA) on the three types of environmental drivers across different DTS fCover ranges **(a-f)**. The figure showed principal component 1 (PC1) vs principal component 2 (PC2) which account for ~64% of the total variance. The arrows in each panel are the loading of different environmental variable from PCA and indicate the direction and strength (length of the arrow) of their contribution to PC1 and PC2. Please see more about the loadings in **Supplementary Figure S7**.

**Figure 7:**
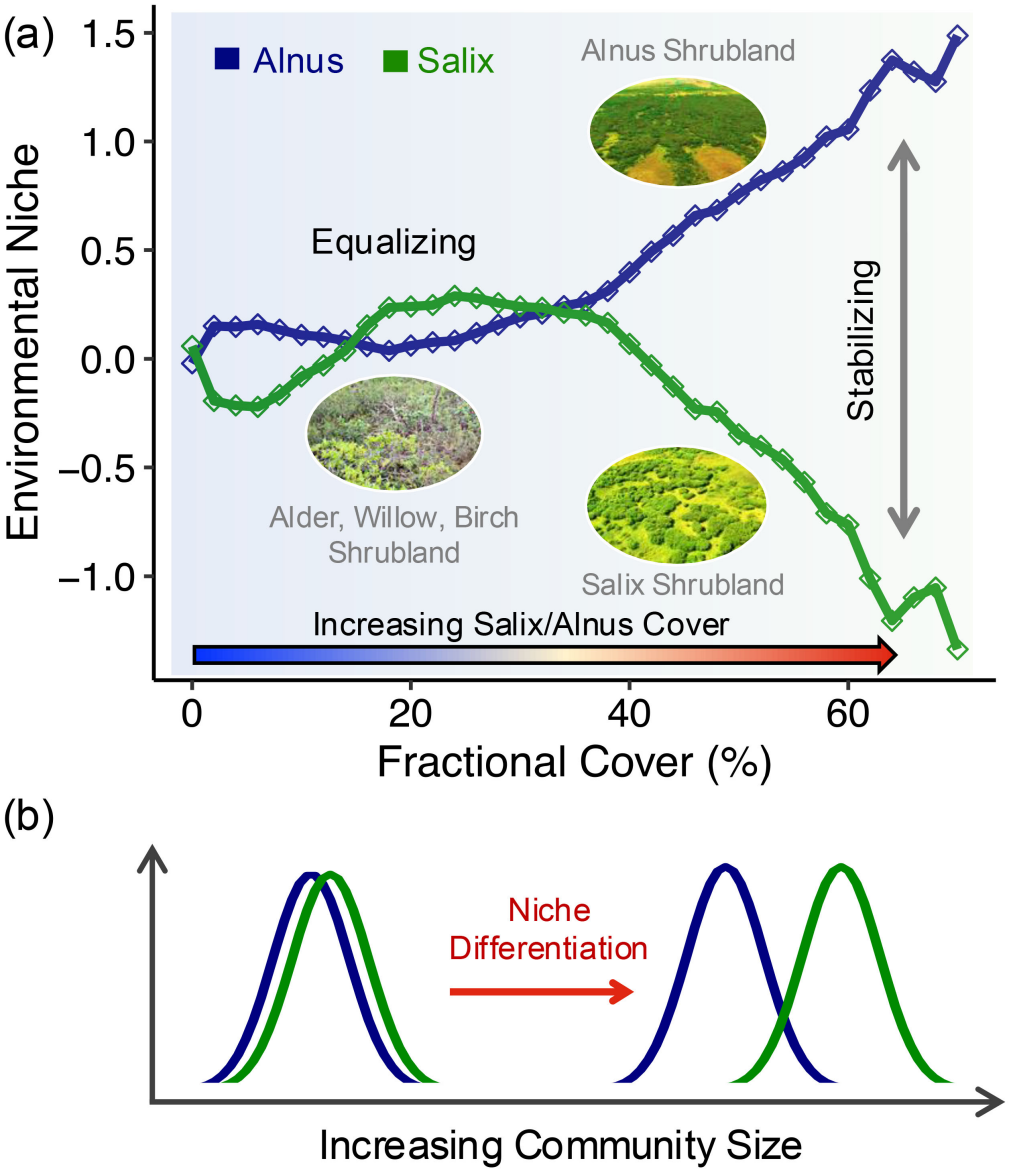
Niche differentiation between *Alnus* and *Salix*. **(a)** Euclidean distance between the centers of *Alnus* and *Salix* in hypervolume environmental space calculated using all 13 environmental variables. The distance shown is relative to the center of the combination of *Alnus* and *Salix* environmental space; **(b)** Illustration of community size effect on niche differentiation between Alnus and Salix, corresponding to the pattern shown in **Figure 7a**. Here ‘equalizing’ in **(a)** indicates that different species tend to share the same resources with weak competition or similar competitive strength among species, while ‘stabilizing’ indicates different species tend to use different resources or space which reduces inter-species competition (Chesson, 2000).

The PCA analysis showed that the first (PC1) and second (PC2) components combined to explain approximately 64% of the variance in environmental drivers regardless of the fCover range included (**Figure 6**). PC1 accounted for about 42% of the variance and was descriptive of energy-related variables (AET, water deficit, ET0, radiation, and T_max_; PC1 in **Supplementary Figure S7**). PC2 was more related to topography and winter climate conditions (elevation, snow, and T_min_) and accounted for about 22% of the variance (PC2 in **Supplementary Figure S7**). See more details about PCA loadings in **Supplementary Figure S7**. In PC1 vs. PC2 space, we observed a high degree of overlap between *Alnus* and *Salix* when pixel communities with DTS cover<30% (i.e., mixed small shrub communities or patches) were used in PCA. However, this overlap decreased significantly, particularly along PC2, when DTS cover became greater than 50% (**Figures 6d, e**), which indicates a potential niche differentiation between large *Alnus* and *Salix* communities likely driven by topography and winter climate gradients. In accordance with PCA, the geometric centers of *Alnus* and *Salix* in hypervolume environmental space were close to each other at small fCover (i.e., small distance between blue and green lines in **Figure 7a**), but diverge for fCover >40%, indicating a potentially “equalizing” mechanism (different species share the same resources and environmental niche) between *Alnus* and *Salix* at small fCover and a “stabilizing” mechanism (niche differentiation among species by resource partitioning where different species rely on different resources) at large fCover (**Figure 7b**).

To understand what drives this differentiation between *Alnus* and *Salix* at larger fCover, we extracted pixels with DTS fCover >50% (i.e., dominated by either *Alnus* or *Salix*) and examined the difference between the two genera under each environmental driver. Overall, six drivers were found to be significantly different between *Alnus* and *Salix*, including slope, TWI, elevation, snow, rain, and T_min_ (**Supplementary Figure S8**). These variables were representative of topography (elevation and slope), water resource distribution (TWI and rain), and winter climate conditions (snow and T_min_), and were mostly related to PC2. However, regions dominated by *Salix* were found to be wetter (higher TWI) and colder (lower T_min_), and have gentler slopes compared to regions dominated by *Alnus*, which was consistent with the above RF analysis that showed a higher *Salix* fCover response at high TWIs and gentle slopes (**Figure 5**).

### 3.4 Functional trait differences between *Alnus* and *Salix*

**Figure 8:**
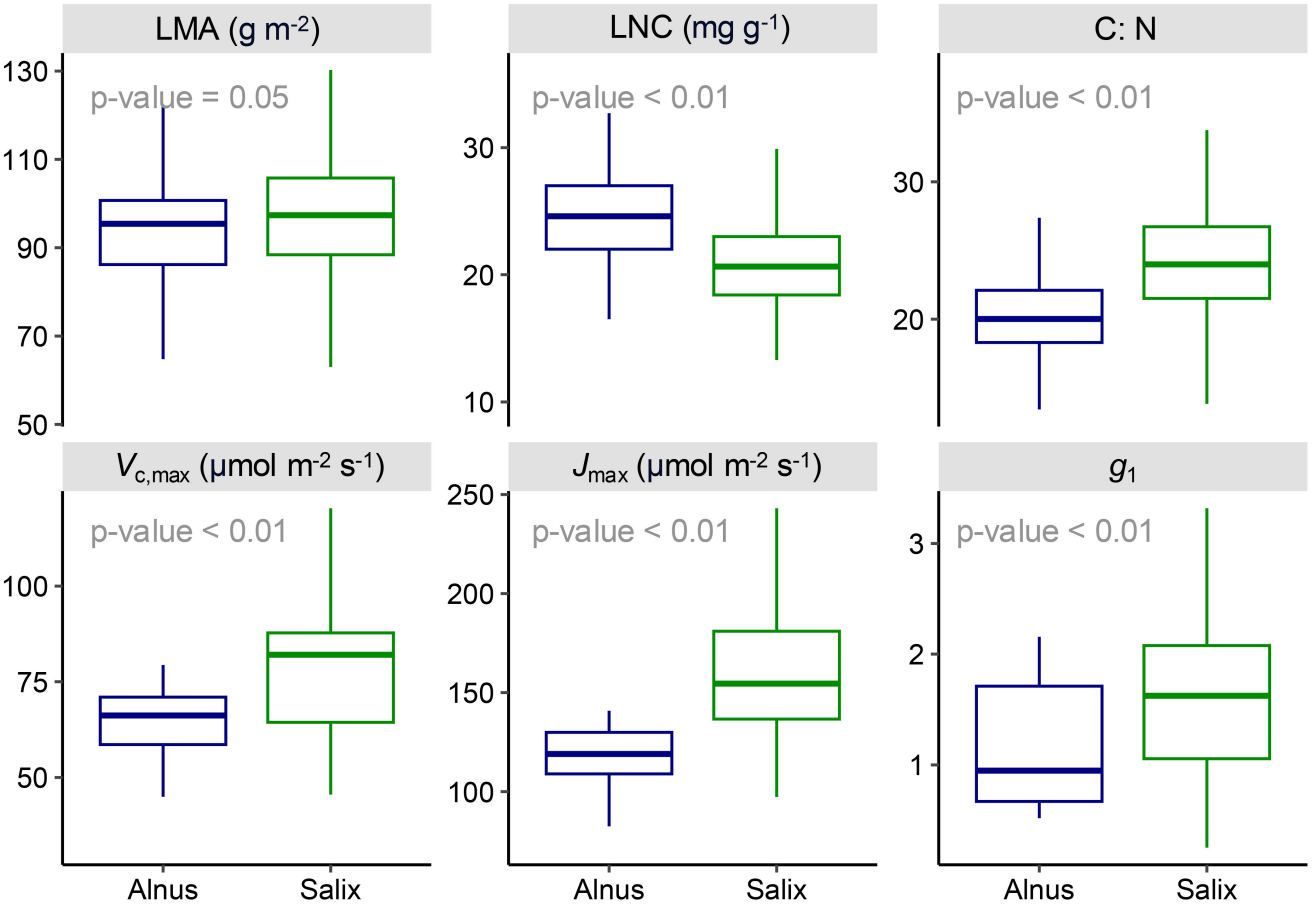
Leaf functional traits of *Alnus* and *Salix* derived from in-site leaf sampling and gas exchange measurements. LMA, leaf mass per area; LNC, leaf nitrogen content; C, N, leaf carbon-nitrogen ratio; *V*_cmax_, maximum photosynthesis capacity at 25 °C, *J*_max_: maximum electron transport rate at 25 °C. *g*_1_: stomatal slope.

The unpaired t-test showed strong differences in most physiological and biochemical traits between *Alnus* and *Salix* (**Figure 8**). In particular, *Salix* was found to have a higher stomatal slope (*g*_1_: *Alnus* 1.02 ± 0.50, *Salix* 1.59 ± 0.72 kPa^0.5^) regardless of community type and soil moisture content (**Supplementary Figure S9**); in other words, lower water use efficiency (WUE) than *Alnus*, as well as higher values for photosynthetic capacity-related traits (*V*_cmax,25_: *Alnus* 63.5 ± 10.5, *Salix*: 76.7 ± 20.9; *J*_max,25_: *Alnus* 116.6 ± 17.8, *Salix* 157.4 ± 37.0 µmol m^-2^ s^-1^). In contrast, *Alnus* had a higher leaf nitrogen concentration (LNC: *Alnus* 24.6 ± 3.5, *Salix* 20.8 ± 3.7 mg g^-1^), and lower C:N ratio (C:N: *Alnus* 20.5 ± 2.9, *Salix* 24.3 ± 4.4), than *Salix*, likely due to its symbiosis with nitrogen-fixing *Frankia* bacteria. However, we did not find a significant difference in leaf morphological traits (LMA: *Alnus* 93.3 ± 12.3, *Salix* 98.7 ± 17.1 g m^-2^) between them, indicating that the two genera might share a similar resource investment strategy for constructing per unit leaf area.

### 3.5 Potential maximum cover and ELFs of *Alnus* and *Salix*

**Figure 9:**
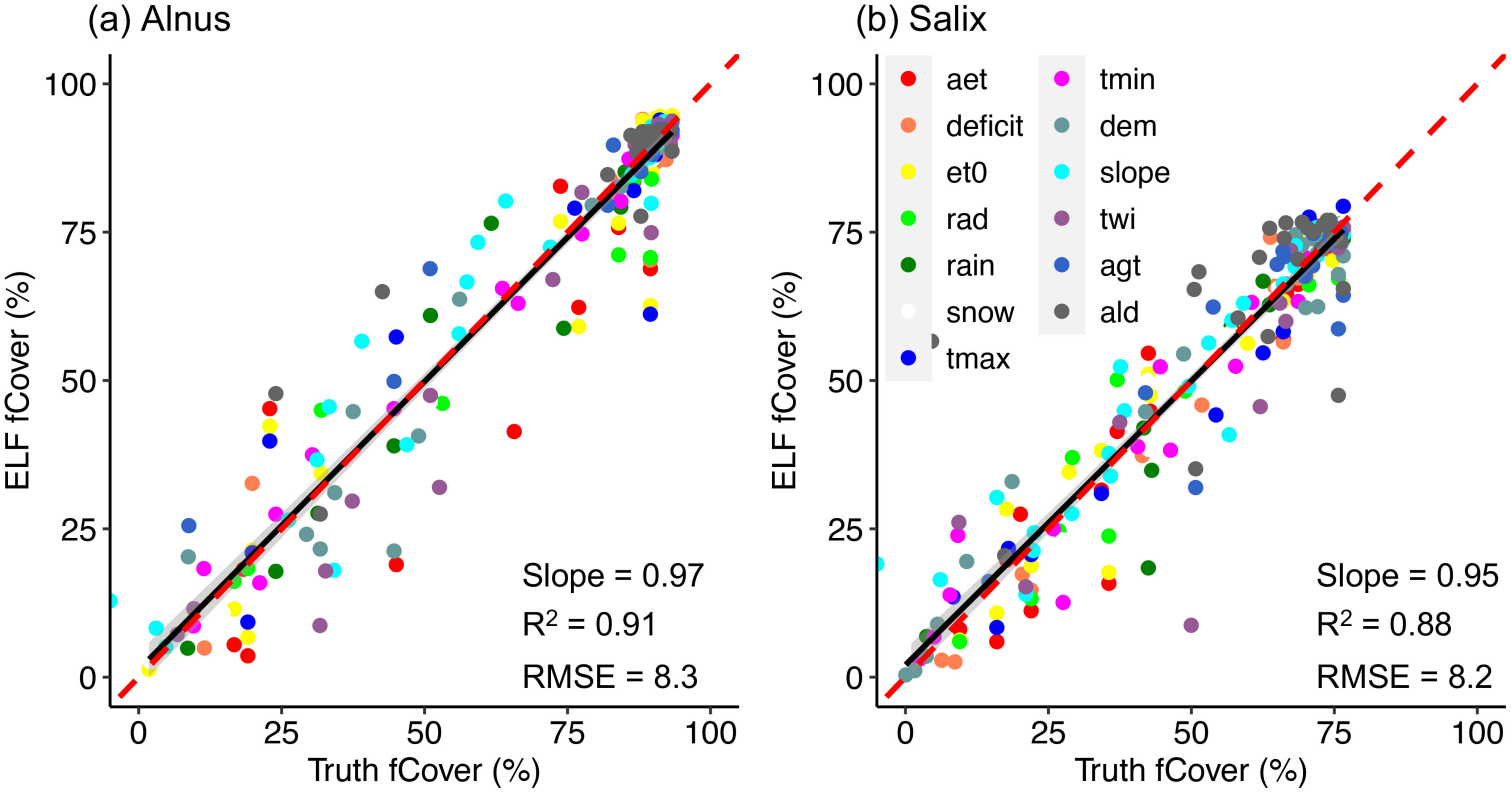
Validation of predicted potential maximum fCover derived from per-variable limiting factor models for *Alnus***(a)** and *Salix***(b)**. The different colors represent different environmental drivers.

We determined the expansion potential of *Alnus* and *Salix* within corresponding ELFs across the landscape using an ELF modeling approach. The per-variable limiting factor models constructed at the 250 m scale well captured the maximum responses (i.e., potential fCover) of *Alnus* and *Salix* fCover to each environment driver (**Supplementary Figures S10** & **S11**), with an overall regression slope >0.95, R^2^ >0.88, and RMSE<8.3 for both genera, when validated against bin-based maximum fCover derived from AVIRIS-NG (**Figure 9**). As expected, the maximum fCover response curves of *Alnus* had much higher peak values (*Alnus*: ~94%) than *Salix* (~77%), consistent with our spatial autocorrelation analysis (**Figure 4**) and suggesting the potential of *Alnus* to form larger, spatially-contiguous communities than *Salix* (**Supplementary Figures S10** & **S11**) under current climatic conditions in the Arctic.

**Figure 10:**
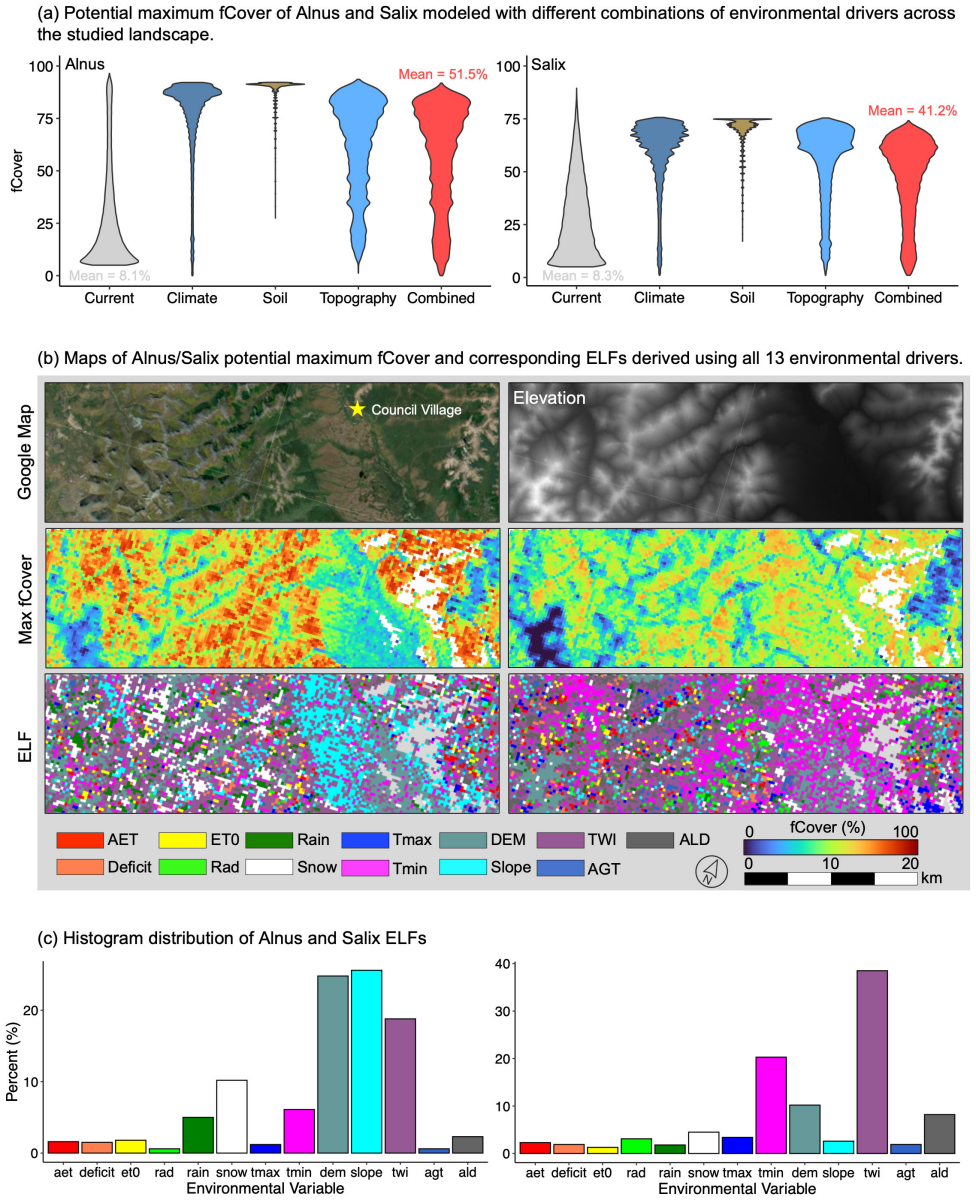
**(a)** Violin distribution of *Alnus* and *Salix* potential fCover predicted across the studied landscape using different combinations of environmental drivers (climate, topography, soil, and the combination of all drivers); **(b)** Maps of the potential *Alnus* and *Salix* fCover constrained using the combination of all environmental drivers and corresponding environmental limiting factor (ELF); **(c)** Histogram distribution of *Alnus* and *Salix* ELFs across different environment drivers.

The potential fCover estimated with climate, topography, and soil constraints, respectively, showed strong differences across the landscape for both *Alnus* and *Salix* (**Figure 10a** & **Supplementary Figure S12**). Notably, climate and soil constraints over-predicted the potential fCover, compared to topographic constraints (elevation, slope, TWI) which yielded the overall lowest potential fCover for both genera (‘topography’ in **Figure 10a** & **Supplementary Figure S12**; mean potential fCover: *Alnus* 56.72 ± 26.87%, Salix 49.63 ± 23.44%) that were close to those modeled using the combination of all 13 environmental constraints (‘combined’ in **Figure 10a** & **Supplementary Figure S12**; mean potential fCover: Alnus 52.02 ± 27.78%, Salix 42.24 ± 22.64%). In the fCover and ELF maps derived using all environmental constraints, we observed high spatial heterogeneity in fCover and corresponding ELFs. For *Alnus*, high potential fCover was generally found on topographic shoulders and backslopes, while lower potential fCover was observed at elevations > 400 m ASL and in the valley near Council. On the other hand, *Salix* showed higher potential fCover on toeslopes and in valleys, but had very low fCover on mountain summits (blue colors in the middle panels of **Figure 10b**). Overall, the main ELFs for *Alnus* (**Figure 10c**) were found to be slope (25.6%), elevation (24.8%), and TWI (18.8%), followed by snow (10.2%) and Tmin (6.1%). In contrast, the dominant ELF for *Salix* (**Figure 10c**) was TWI (38.5%), followed by Tmin (20.3%), elevation (10.2%), and ALD (8.2%). However, we also observed that the dominant ELFs change with potential fCover. For example, while regions with low *Alnus* potential fCover (<25%) were strongly constrained by low elevation and slope, low TWI was a dominant constraint for regions where large *Alnus* fCover (>50%) can develop (**Supplementary Figure S13a**). Similarly, *Salix* was increasingly constrained by low T_min_ and high elevation in regions with large potential fCover, compared to regions with low *Salix* fCover which were dominantly constrained by low TWI (**Supplementary Figure S13b**).

## 4 Discussion

In this study, we investigated the patterns and primary controls of the distribution of *Alnus* and *Salix*, two key DTS genera, in the low-Arctic tundra of Alaska’s Seward Peninsula. We used multi-scale, multi-platform remote sensing data, modeled soil condition information, climate observations, and *in situ* leaf trait measurements to help spatially discern the fCover of *Alnus* and *Salix*, and explore their relationships with environmental and biological drivers. We showed that topographic features played an important role in controlling the contemporary distribution of DTSs, creating heterogeneous but predictable distributions of their fCover. We also observed strong spatial differences in the distribution and niche of *Alnus* and *Salix*, associated with environmental controls and leaf functional traits. In particular, physiological traits related to resource acquisition (*V*_cmax_, *J*_max_) and water use efficiency (*g*_1_) were significantly different between *Alnus* and *Salix*, likely playing a critical role in the niche differentiation between the two genera.

### 4.1 Topography-controlled processes drive overall DTS distribution and differences between *Alnus* and *Salix*

We first sought to understand the general spatial patterns and drivers of DTS distribution. We observed that the abundance (i.e., fCover) of *Alnus* and *Salix* was highly variable across the landscape, mirroring the heterogeneous nature of DTS distribution in the Arctic (Tape et al., 2012; Frost et al., 2013). This spatial heterogeneity was strongly driven by topographic gradients, including elevation, slope, and TWI, and differed between *Alnus* and *Salix* (**Figure 5**). In particular, the distribution of *Alnus* was highly sensitive to elevation and slope, with high *Alnus* fCover concentrated on shoulders and backslopes (**Figure 2**) with steep surfaces (10 - 25°) and lower TWIs (6 - 8) and within an elevational band of 200–360 m. From our analysis, it remains unclear which biophysical processes associated with this elevational band drive these patterns. Previous studies have shown that shallow soil layers, rocky soil and ground, and more summer warmth in uplands lead to a high frequency of cryoturbation (e.g., frost boils; Iturrate-Garcia et al., 2016), which exposes mineral soils that favors the recruitment and growth of *Alnus* (Sutton et al., 2006; Virtanen et al., 2010; Frost et al., 2013). At the same time, relatively drier, unsaturated, and silt-rich soils on hillslopes (**Supplementary Figure S9**) provide better growth conditions for free-living *Frankia* bacteria and *Alnus* roots which do not appear to thrive in saturated soils (Tape et al., 2012). In contrast to *Alnus*, the distribution of *Salix* was strongly driven by TWI, an indicator of soil moisture, with larger *Salix* cover at sites with high TWIs (e.g., riverbanks and drainages) and gentle slopes (3 - 10°). This finding is consistent with previous studies (Boulanger-Lapointe et al., 2014) and suggests that topography-controlled hydrological processes play an important role in determining the expansion of *Salix*. In this study, we showed that *Salix* had a higher stomatal slope compared to *Alnus* (*g_1_*: 1.59 ± 0.72 for *Salix*; 1.02 ± 0.5 for *Alnus*; **Figure 8**). This high stomatal slope indicates that *Salix* is more profligate with respect to its water use (i.e., lower WUE). In other words, the growth of *Salix* is more water demanding, which likely contributes to its distribution in regions with higher TWIs.

In addition to high heterogeneity, we also showed that *Alnus* could form much larger patches than *Salix* (radius: 750 m for *Alnus*; 250 m for *Salix*) under favorable growth conditions (**Figure 4**). This is likely because the nitrogen fixation of *Alnus*-*Frankia* symbiosis enables them to better colonize nutrient-poor environments (Salmon et al., 2019; Chen et al., 2020; Schore et al., 2023), which promotes the spatial expansion of *Alnus*. Such symbiosis also increases soil nutrient availability, which, combined with faster litter decomposition (especially in winter seasons) and altered micro-environments (e.g., light, temperature, and hydrology; Yang et al., 2021), creates a positive feedback loop that could enhance the ‘infilling’ and range expansion of *Alnus* communities (Sturm et al., 2005). Additionally, while phosphorus co-limits vegetation growth in the Arctic (Devotta et al., 2021; Salmon et al., 2019), exposed soils and rocks provide better phosphorus availability on upper slopes, which could facilitate the expansion of *Alnus*.

Collectively, these findings demonstrate that *Alnus* and *Salix* vary significantly in their spatial distribution at regional scale driven by topographic and moisture gradients, thus responding to climate change differently. Understanding these differences among genera is important to predict future shrub expansion and its impacts on Arctic ecosystems. For instance, an increase in *Alnus* cover (high leaf nitrogen content; **Figure 8**) in highlands could significantly increase downslope nitrogen availability through hillslope hydrology, driving further changes in vegetation and ecosystem carbon storage (McCaully et al., 2022). On the other hand, *Salix* expansion will likely have stronger impacts on ecosystem water cycling with more profligate water use, potentially altering land-atmosphere interactions (Loranty and Goetz, 2012). It should be noted that, although we showed topographic position (elevation and slope) and topography-controlled hydrological processes (TWI) are strong determinants of *Alnus* and *Salix* distribution (collectively explain 82.5% and 79.4% the variance in *Alnus* and *Salix* fCover variation respectively; **Supplementary Figure S14**), topographic gradients tend to co-vary with climate variables−for example, air temperature typically decreases with elevation and solar radiation varies with aspect (Raupach and Finnigan, 1997). Therefore, the observed significance, particularly for elevation, may reflect the combined effects of both topography and climate. Yet, the higher VIPs of elevation compared to any individual climate variable suggests that elevation captured additional fine-scale ecological processes not represented in climate data, such as soil type, nutrient distribution, geology, and micro-scale disturbances (Soest et al., 2025; Abolt et al., 2024). Future efforts will be needed to identify functional relationships between topography and ecological properties to better capture the fine-scale processes that regulate the establishment and distribution among these species in order to better forecast how climate change will alter the distribution of important shrub species in the Arctic.

### 4.2 Community size and physiological traits affect niche differentiation between *Alnus* and *Salix*

We found that the niche differentiation between *Alnus* and *Salix* depended on patch size (**Figures 6** & **7**). Both genera appeared to share similar environments when community/patch size was small (fCover< 40%), but this similarity decreased with increasing patch size and began to diverge as fCover approached 40%. This pattern indicates a potentially “equalizing” mechanism (i.e., different species share the same resources with weak competition or similar competitive strength among species; Chesson, 2000) that promotes the co-existence of individuals or small patches of *Alnus* and *Salix* (e.g., alder-willow-birch shrubland; **Supplementary Figure S6**), but a “stabilizing” mechanism (i.e., different species tend to rely on different resources or space which reduces inter-species competition; Chesson, 2000) that separates the development of large *Alnus*/*Salix* communities. In other words, large communities have more specialized resource requirements than individual plants or small patches of *Alnus* and *Salix* (**Figure 7b**), leading to their spatial separation, such as the observed transition in shrub dominance from *Salix* to *Alnus* when traveling from west to east of the Seward Peninsula (**Figure 2**). Currently, studies of shrubification in the Arctic have focused on large communities (e.g., alder or willow shrubland) that can be directly identified from coarse remote sensing observations (e.g., Landsat). The mechanisms that drive the co-existence of *Alnus* and *Salix* and its impacts on local biodiversity and biogeochemical cycling remain significantly understudied, despite such coexistence having been broadly observed across the Arctic (e.g., alder-willow-birch shrubland; **Supplementary Figure S6**). In addition to the equalizing mechanism, several processes may also contribute to the co-existence of *Alnus* and *Salix*. For example, nitrogen inputs from nitrogen-fixing *Alnus* have been shown to have a nursing effect on other plant types, likely contributing to the establishment of *Salix* in open-canopy alder communities or on downslopes where nitrogen availability is enriched by upper-slope *Alnus* (Schore et al., 2023). Moreover, high spatial variation in environmental conditions can create micro-scale habitats that facilitate the inter-dispersed establishment of *Alnus* and *Salix*. Our future studies will explore this mechanism by integrating ground observations and fine-scale surface and belowground measurements from UASs.

For large *Alnus*/*Salix* communities (fCover >50%), five primary environmental variables contributed to their niche differentiation: slope, TWI, T_min_, snow, and rain (**Supplementary Figure S8**). These variables capture the impacts of topography, moisture, and winter climate conditions. As expected, regions dominated by *Alnus* were found to have lower soil moisture (TWI), steeper slope, and warmer winter temperature (T_min_) than regions dominated by *Salix* (**Supplementary Figure S8**). This finding is consistent with our DTS cover analysis (discussed in section 4.1) and suggests that *Salix* thrives in regions with higher soil moisture and gentler slopes than *Alnus*. In addition, the lower T_min_ of *Salix* implies that they are more tolerant of colder environments compared to *Alnus*, and thus more likely to expand into the northern Arctic region with climate warming (Boulanger-Lapointe et al., 2014). However, we did not find elevation (i.e., DEM) to be significantly different between large *Alnus* and *Salix* communities (p>0.1; **Supplementary Figure S8**), as contrary to its role in driving the overall spatial variation in DTS fCover. This is likely because both *Salix* and *Alnus* shrublands distributed on hillslopes across a large topographic gradient (5–300 m) that blurred their separation along DEM. On the other hand, it indicates that separation between large *Alnus* and *Salix* is more sensitive to relative topographic position, instead of absolute DEM.

In alignment with the different sensitivities of *Alnus* and *Salix* to moisture conditions (TWI), our trait measurements found that *Salix* had an overall lower WUE (higher *g*_1_) than *Alnus* (**Figure 8**), manifesting a potential biological mechanism that drives their niche differentiation along a soil moisture spectrum. We broke down *g*_1_ and associated soil moisture data (see 2.5) into four shrub community types and showed that *g*_1_ is lower for *Alnus* regardless of living environments (i.e., soil moisture) or community type (**Supplementary Figure S9**). This suggests that environmental filtering on plant traits likely plays an important role in shaping shrub distribution in the Arctic (Myers-Smith et al., 2018). This analysis also confirmed that soils in *Alnus* shrubland are significantly drier than those in *Salix* communities (willow shrubland and willow birch shrubland in **Supplementary Figure S9**) and where *Alnus* and *Salix* coexist (alder, willow, birch shrubland in **Supplementary Figure S9**). In addition to *g*_1_, *Salix* also had a higher photosynthetic capacity (*V*_cmax,25_: 76.7 ± 20.9; *J*_max,25:_ 157.4 ± 37.0 µmol m^-2^s^-1^) compared to *Alnus* (*V*_cmax,25_: 63.5 ± 10.5; *J*_max,25:_ 116.6 ± 17.8 µmol m^-2^s^-1^), indicative of a greater potential for carbon assimilation. However, from this study, it is not clear how photosynthetic traits affect niche differentiation between *Alnus* and *Salix*. It should be noted that physiological measurements remain critically lacking in the Arctic (Rogers et al., 2021). We recommend future studies focusing on collecting or synthesizing plant physiological data in the broader Arctic region to understand the biological differences between *Alnus* and *Salix*, as well as their role in regulating DTS distribution.

### 4.3 Topography influences the upper bound of the climate-driven expansion of *Alnus* and *Salix* cover

Shrub expansion is not ‘unconditional’ in the Arctic and ultimately will be limited by abiotic or biotic (e.g., species competition) factors that do not favor their growth (Swanson, 2015; Schore et al., 2023). Previous studies have focused on identifying the drivers that promote overall shrub expansion, e.g., summer temperature and permafrost thaw (Boyle et al., 2022; Chen et al., 2020; Andreu-Hayles et al., 2020). In this study, we showed that increases in DTS cover across our studied landscape were more likely constrained by topographic conditions (e.g., elevation, slope, and TWI; **Figure 10** and **Supplementary Figure S14**). In other words, despite the widespread climate-driven shrub expansion (either through infilling or range spread), the suitability of an area for DTSs and their maximum fCover may ultimately be governed by topography and associated regional processes not captured by climate variables, such as soil moisture, soil type, rock layer, and nutrient distribution, particularly in regions with high topographic gradients. In those regions, tall shrubs must overcome the ‘adversity’ posed by topographic constraints, potentially through changes in disturbance regimes, hydrology, and permafrost dynamics (Mekonnen et al., 2021a). This once more highlights the need to capture topographic controls in dynamic vegetation models to improve the prediction of shrub changes in the Arctic.

We also showed that the potential of DTS expansion and corresponding ELFs were highly variable across the landscape because of the high spatial variability in topography and micro-climate conditions (Virtanen and Ek, 2014; Wu et al., 2020), and also differed significantly between *Alnus* and *Salix.* Low or high elevations and low slopes appeared to be the main limiting factor for *Alnus* at low abundance (potential fCover<25%; **Supplementary Figure S13a**). This is likely because the rocky and nutrient-poor environments at higher summits or saturated soils in valleys and on flat surfaces tends not to favor the development of *Alnus* seedlings (Tape et al., 2012; Salmon et al., 2019). However, low soil moisture conditions (TWI) became increasingly limiting for regions with higher *Alnus* expansion potential (potential fCover >50%), potentially because increased transpiration in dense shrub communities has higher water demand (Bring et al., 2016). In comparison, regions with low *Salix* expansion potential were primarily limited by low TWI (**Supplementary Figure S13b**), due to their high sensitivity to soil moisture during seed generation (Boulanger-Lapointe et al., 2016) and proliferate use of water observed in this study (low WUE). But, winter temperature (T_min_), elevation, and permafrost conditions (ALD), emerged to be considerable constraints for the further development of large *Salix* communities, which indicates that the generally harsh environment of the Arctic could partially offset hydrology-controlled enhancement (e.g., increasing soil moisture caused by snowmelt and increased precipitation; McCrystall et al., 2021) on *Salix* expansion (Boulanger-Lapointe et al., 2016; Chen et al., 2021; Jin et al., 2021).

It should be noted that we did not find a significant limit of soil properties (ALD and AGT) on DTS expansion, which have been shown to be important determinants of plant community composition (Standen and Baltzer, 2021). From this study, we cannot tell if this is because their spatiotemporal variation is strongly coupled with topographic and climate variables (Léger et al., 2019), and thus their impacts are ‘buried’ in variables that appeared to be stronger constraints on DTSs, such as elevation and slope. Also, soil processes may influence vegetation distribution at scales (<1 m; Suvanto et al., 2014; Niittynen et al., 2020b) much smaller than what was examined in this study (250 m). This scale mismatch may be more pronounced given the original coarser resolution (500 m) of ALD and AGT data from Debolskiy et al. (2019). Future studies may explore this by combining ground-based measurements with high-resolution remote sensing, such as UAS (Assmann et al., 2020; Yang et al., 2022). Lastly, soil processes are successively affected by vegetation distribution (Lawrence and Swenson, 2011; Kropp et al., 2020), which makes it challenging to disentangle the cause-effect relationships between permafrost-soil properties and shrub growth.

### 4.4 Caveats and next steps

While we demonstrated the potential of multi-scale, multi-platform remote sensing for understanding Arctic shrubification, we also identified several limitations that could be improved in future studies. First, despite the fact that we used a sizable study area (42 × 18 km) that was representative of *Alnus* and *Salix* distribution in western Alaska, the spatial extent of the studied landscape is still relatively limited and cannot represent the full breadth of DTS distribution across the Arctic. As a result, the observed patterns may not be the case for other ecoregions, such as the Canadian Arctic or Arctic Russia. Future studies will combine UAS (e.g., High-Latitude Drone Ecology Network [HILDEN]), AVIRIS-NG (Miller et al., 2019), and satellite data collected across broader areas (e.g., the ABoVE domain) to validate and expand our analyses. Second, we investigated DTS distribution at a scale (250 m) that maximized the correlation between environmental factors and DTS cover, but different environmental factors may influence shrub distribution at different scales. For example, soil moisture may affect plant distribution at much smaller scales (<1 m; Suvanto et al., 2014) than air temperature. This may lead to slight biases in the observed importance of some environmental variables, such as soil properties. However, such biases should not affect the overall patterns revealed in this study, given the observed consistency of our findings across different analyses and with previous studies (Mekonnen et al., 2021b). Future studies may improve this by incorporating higher resolution data (e.g., 2 m ArcticDEM (Porter, 2023) and 30 m airborne SAR-derived soil products (Rosen et al., 2017)) to disentangle the impacts of different factors. Third, this study focused on exploring the environmental limits of shrub expansion, which is a key concern with continued climate change (Myers-Smith et al., 2011a; Mekonnen et al., 2021a). Other factors, like parent material, species competition, and disturbances, could also affect the realized distribution of DTSs (Chapin et al., 1989; Frost et al., 2013; Salmon et al., 2019; Chen et al., 2021), but will be hard to quantify with remote sensing methods. In future analysis, we will incorporate field surveys, existing and continued collection of UAS data (e.g., HILDEN), as well as site disturbance history to investigate the impacts of these factors on the expansion of DTSs. In addition, while we show topography and associated processes are strong controls of DTS distribution, the impacts of topography are complicated and much beyond elevation, slope, and TWI, like geology, disturbance, and nutrient distribution which was not explored in this study due to lack of relevant data. This study showed topography as a useful proxy of local climate and other variables that are hard to capture with remote sensing data, such as soil type and nutrient distribution, but future analysis is needed to better understand the relationships between topography and other ecological properties for predicting shrub distribution in the Arctic, as well as how these relationships impact understanding and modeling vegetation dynamics which has been historically difficult to decouple in remote sensing data and ecological analysis in general (Feng et al., 2019). Lastly, we illustrated that our observed niche differentiation between *Alnus* and *Salix* is also associated with their physiological differences in leaf water use efficiency (**Figure 8** & **Supplementary Figure S9**). However, more measurements should be made to examine the physiological differences between *Alnus* and *Salix.* Building on this finding, we will expand the scope of this study to include other functional traits, such as leaf phenology, morphology, and biochemistry. For example, incorporating high-resolution phenology observations from PlanetScope (Wang et al., 2020) will allow us to characterize phenological differences between plant species across space to better understand the observed different distributions of *Alnus* and *Salix*.

In conclusion, this study provided valuable insights into the patterns, drivers, and limits of DTS (*Alnus* and *Salix*) distribution in the Arctic. First, topography plays an important role in controlling the distribution and future expansion of DTSs, creating a heterogeneous but predictable distribution in *Alnus* and *Salix* cover. Second, different shrub species differ significantly in spatial distribution and environmental niche, associated with their differences in primary controlling factors and functional traits (e.g., physiology). Third, niche differentiation between *Alnus* and *Salix* is influenced by community size, with larger communities being more specialized in resource requirements than individual plants or small patches of *Alnus* or *Salix*. Fourth, topography, and associated hydrological processes, likely control the upper bound of the climate-driven expansion of *Alnus* and *Salix* cover. Collectively, these findings highlight a critical need to 1) improve our ecological understanding and model representation of topography-controlled processes and functional traits in regulating shrub distribution and 2) develop more detailed species characterization of shrub distribution, dynamics, and drivers to better understand shrub responses to climate change.

## Data Availability

The datasets presented in this study can be found in online repositories. The names of the repository/repositories and accession number(s) can be found below: https://data.ess-dive.lbl.gov/view/doi%3A10.15485%2F2441506, https://data.ess-dive.lbl.gov/view/doi%3A10.15485%2F2335763.
