# Supplementary material for: Topography and functional traits shape the distribution of key shrub plant functional types in low-Arctic tundra
Source: Front Plant Sci. 2026 Jan 7;16:1724838. doi: 10.3389/fpls.2025.1724838 (PMC12819713; doi:10.3389/fpls.2025.1724838)
Supplement: Supplementary file 1 [file DataSheet1.pdf]

Journal: Frontiers in Plant Science

Title: Topography and Functional Traits Shape the Distribution of Key Shrub Plant Functional Types in Low-Arctic Tundra

Author List: Daryl Yang[1,2,3*], Wouter Hantson[4,5], Kenneth J. Davidson[2,3,6], Julien Lamour[7], Bailey D. Morrison[8], Verity G. Salmon[1], Tianqi Zhang[1], Kim S. Ely[2,9], Charles E. Miller[10], Daniel J. Hayes[4], Stephen Baines[3], Alistair Rogers[2,9], Shawn P. Serbin[2,3,11]

Author Affiliation:

1. Environmental Sciences Division and Climate Change Science Institute, Oak Ridge National Laboratory, Oak Ridge, TN, USA
2. Environmental and Climate Sciences Department, Brookhaven National Laboratory, Upton, NY, USA
3. Department of Ecology and Evolution, Stony Brook University, Stony Brook, NY, USA
4. School of Forest Resources, University of Maine, Orono, ME, USA
5. WSL Institute for Snow and Avalanche Research SLF, Alpine Environment and Natural Hazards, Davos Dorf, Switzerland
6. American Forests, Washington DC, USA
7. Centre de Recherche sur la Biodiversité et l'Environnement (CRBE), Université de Toulouse, CNRS, IRD, Toulouse INP, Université Toulouse 3 – Paul Sabatier (UT3), Toulouse, France
8. Department of Civil and Environmental Engineering, University of California, Merced, Merced, CA, USA
9. Climate and Ecosystem Sciences Division, Berkeley National Laboratory, Berkeley, CA, USA
10. Jet Propulsion Laboratory, California Institute of Technology, Pasadena, CA, USA
11. Biospheric Sciences Laboratory (Code 618), NASA Goddard Space Flight Center, Greenbelt, MD, USA

***Corresponding author:** Daryl Yang (email: yangd@ornl.gov)

Contents of this file:
Figure S1 to S14
Table S1

Introduction

This document includes 12 supplementary figures and 1 supplementary table to support results and findings presented in "*Topography and Functional Traits Shape the Distribution of Key Shrub Plant Functional Types in Low-Arctic Tundra*" submitted to *Frontiers in Plant Science*. The raw data and methods for producing these figures and table, and the interpretation of these results, are fully presented in the submitted manuscript.

Fig. S1: Uncertainty of *Alnus* and *Salix* fCover derived from Airborne Visible / Infrared Imaging Spectrometer Next Generation (AVIRIS-NG). The uncertainty histograms were calculated using the entire map uncertainty map of *Alnus* and *Salix*.

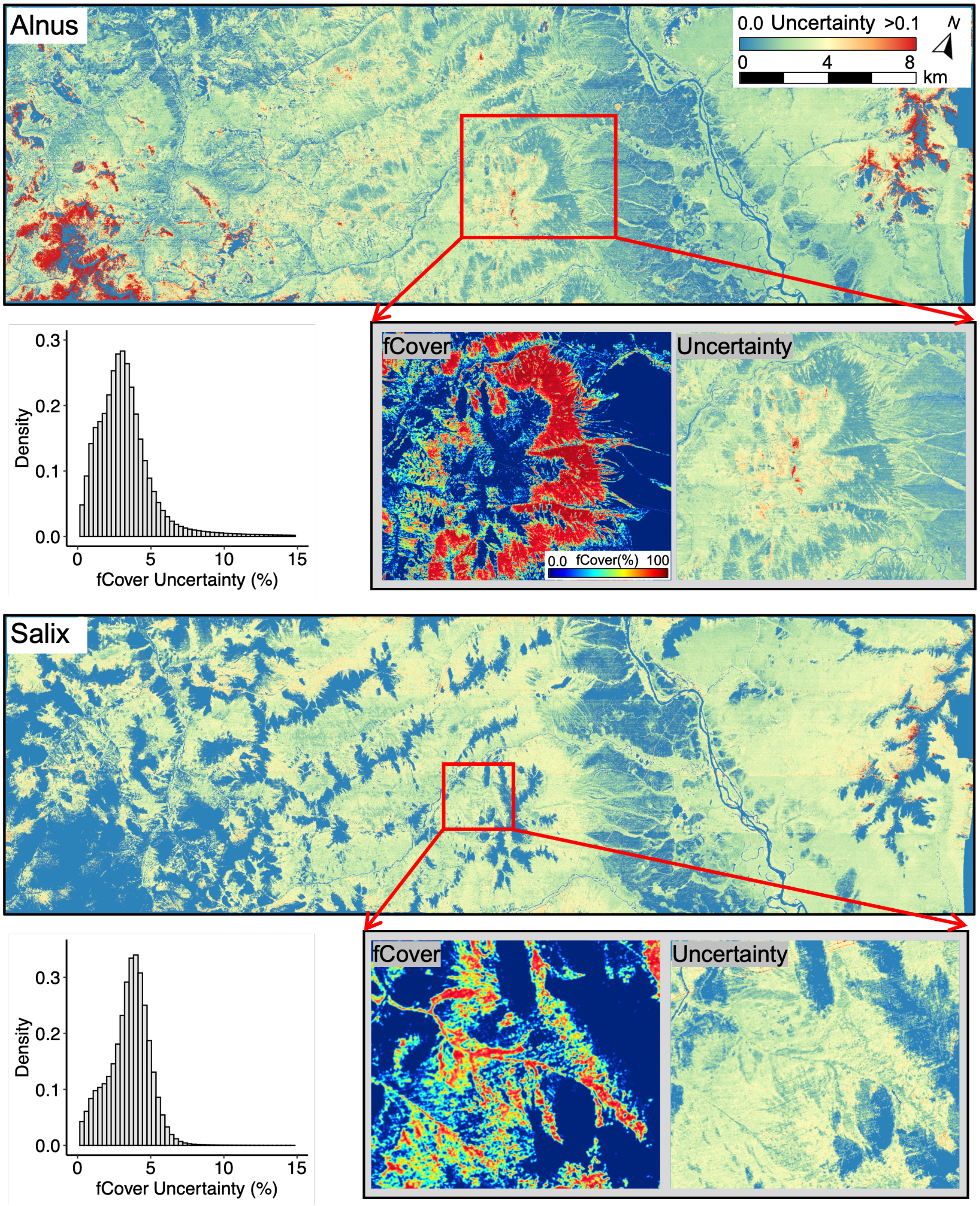

Fig. S2: Red-green-blue (RGB) imagery, canopy height model (CHM), and classification maps derived from very-high-resolution (5 cm) Unoccupied Aerial System (UAS) imagery.

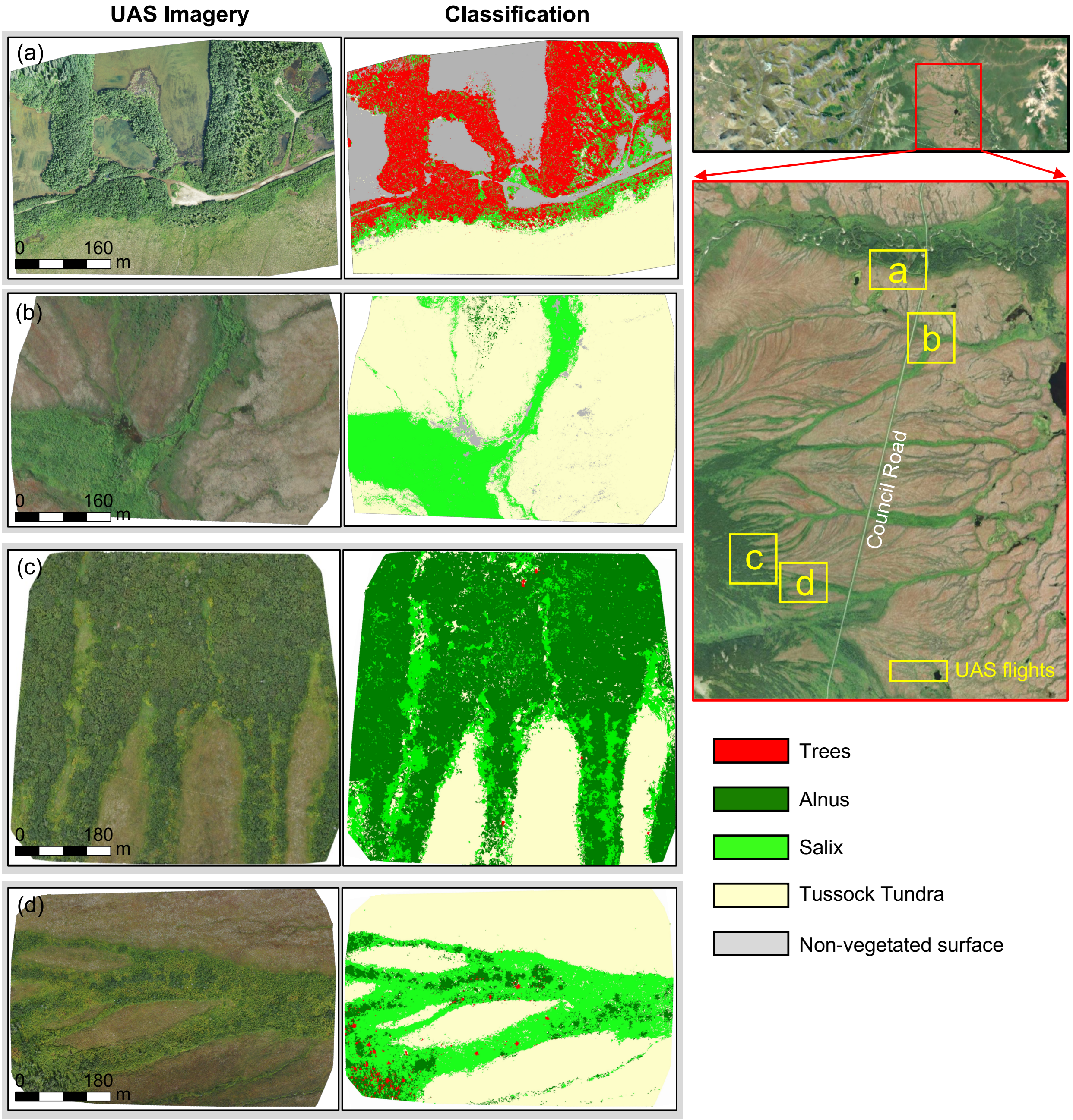

Fig. S3: Environmental drivers used in this study. Climate drivers: actual evapotranspiration (AET), potential evapotranspiration (ET0), water deficit (i.e., ET0 - AET), radiation, rain, snow, annual maximum temperature (T_{max}), and annual minimum temperature (T_{min}); Topographic drivers: elevation, slope, and topographic wetness index (TWI); Soil drivers: active layer depth (ALD), annual ground temperature (AGT).

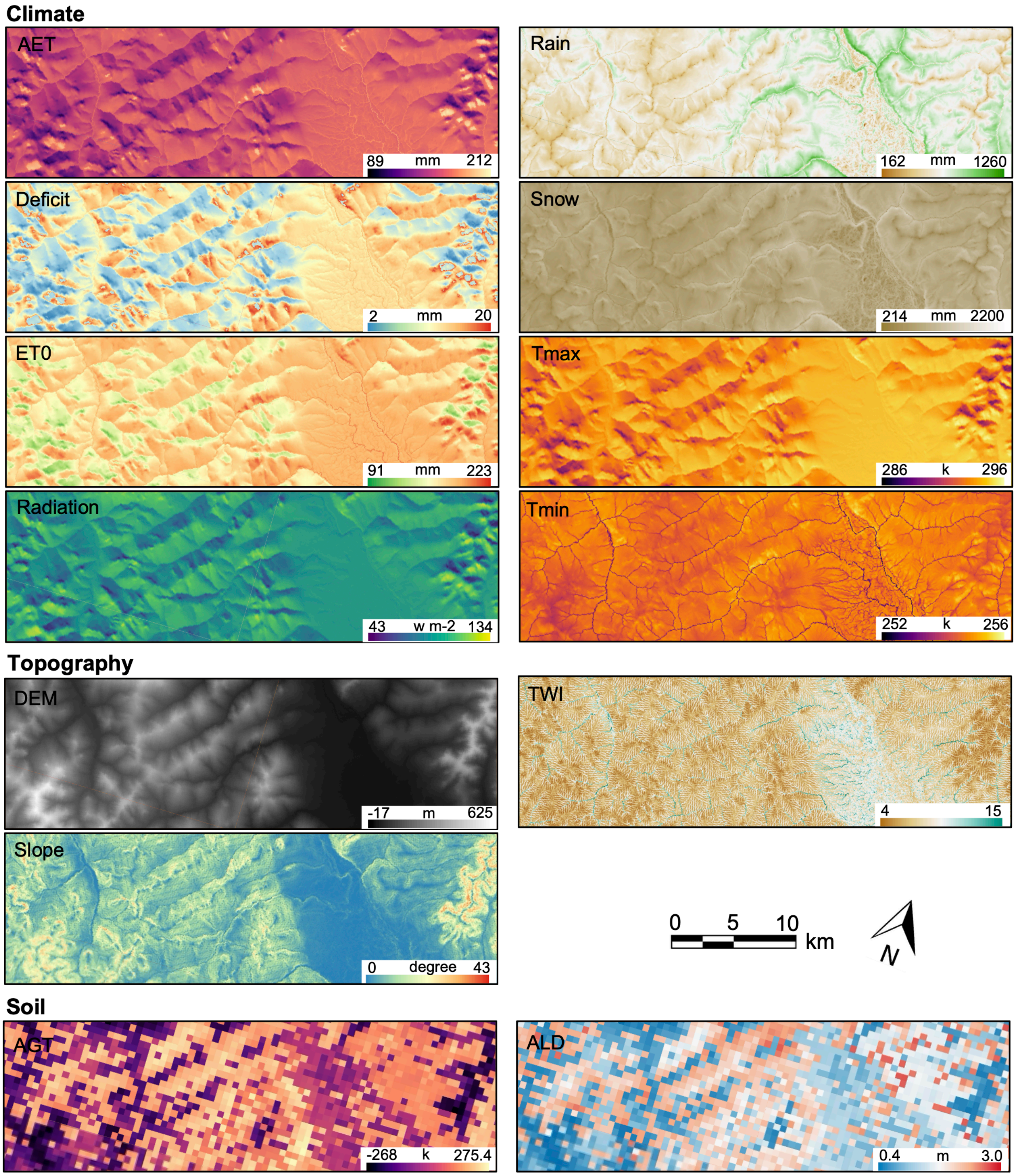

Fig. S4: Variance in the spatial variation in fCover explained by environmental drivers. The best scale (250 m) for modeling *Alnus* and *Salix* fCover is determined as the one that rendered the highest variance explained.

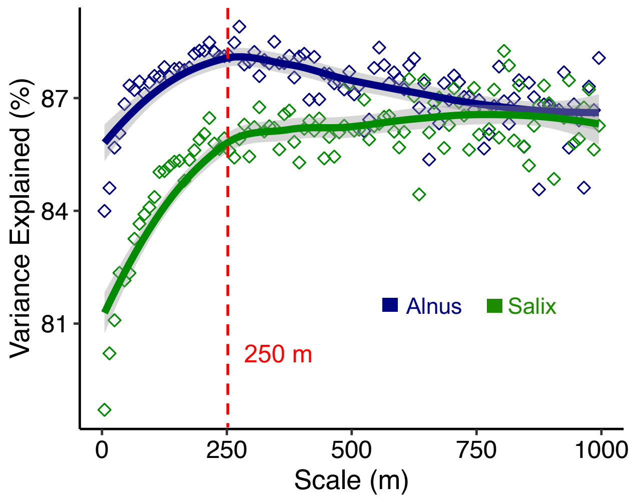

Fig. S5: Comparison of variable importance for projection between RF models constructed with all predictor variables (a) vs. RF models after removing variables with high inflation factor values (b). Removing variables (i.e., aet, deficit, et0) with high variable inflation factor (VIF, c) had little impacts on the overall distribution and ranking of predictor variables. Thus, this study included all 13 predictor variables in our RF models.

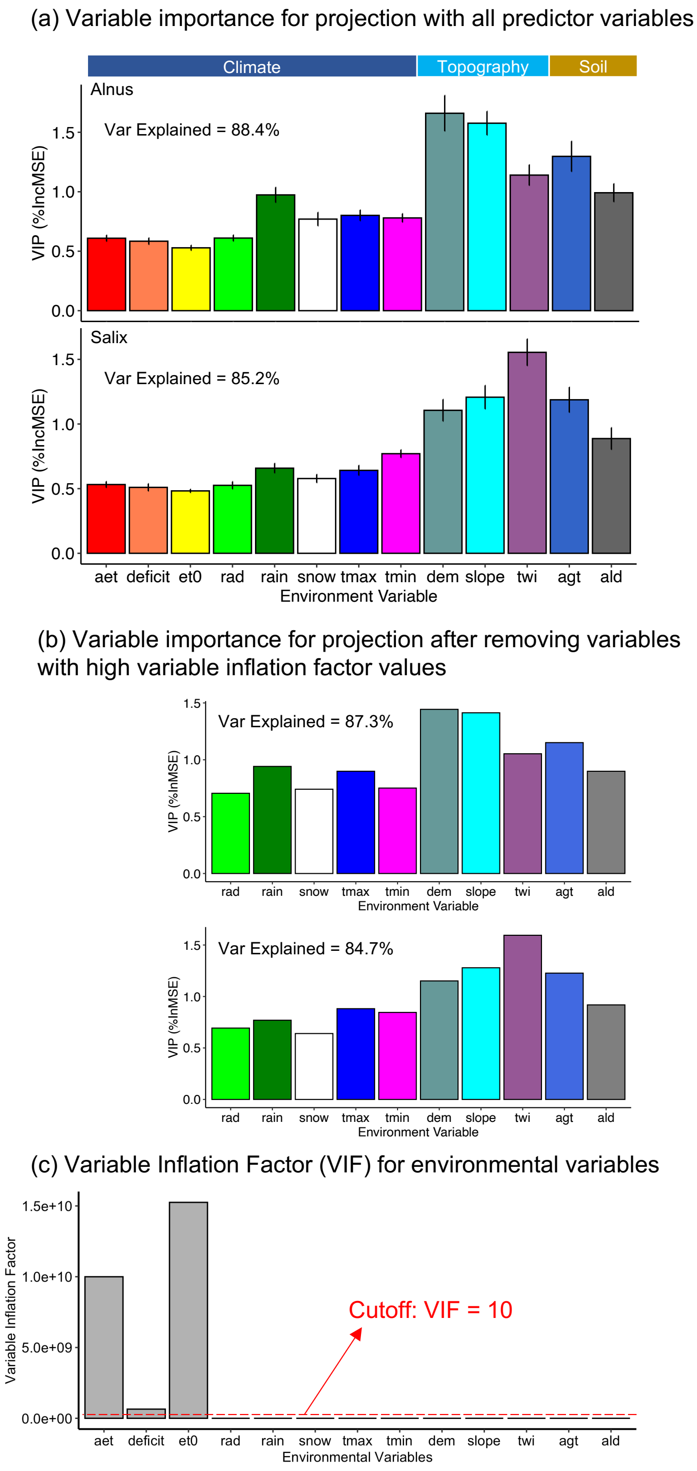

Fig. S6: Examples of mixed *Alnus* and *Salix* across the landscape. Imagery used for this figure is from ESRI world imagery. Inset (a), (b), and (c) are from imagery collected on September 5, 2015. Inset (d) is from imagery collected on July 9, 2019. *Salix* is observed as the yellowish color in (a), (b), and (c), and light green color in (d). *Alnus* is observed as the dark green color in all four insets.

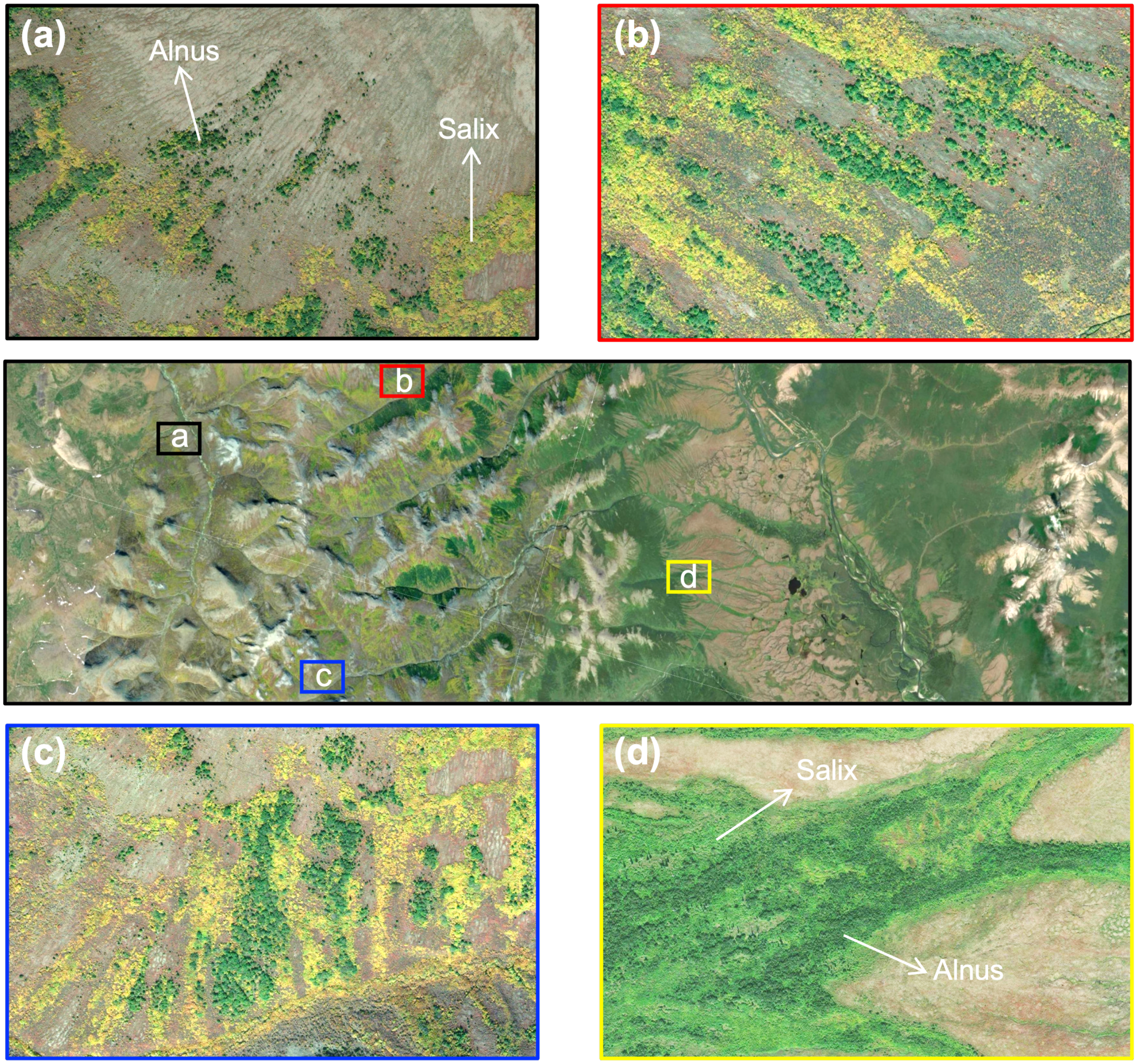

Fig. S7: Loading from principal component analysis (PCA). Here, we show loading for both fCover >50% and 60%, which demonstrated consistent patterns. PC 1 and PC 2 explained about ~66% of the variance in the environmental driver variation of *Alnus* and *Salix*. PC1 is more descriptive of energy related variables (e.g., AET, deficit, ET0, radiation, and Tmax). PC 2 is more related to topography and winter climate conditions (e.g., snow, DEM, and Tmin)

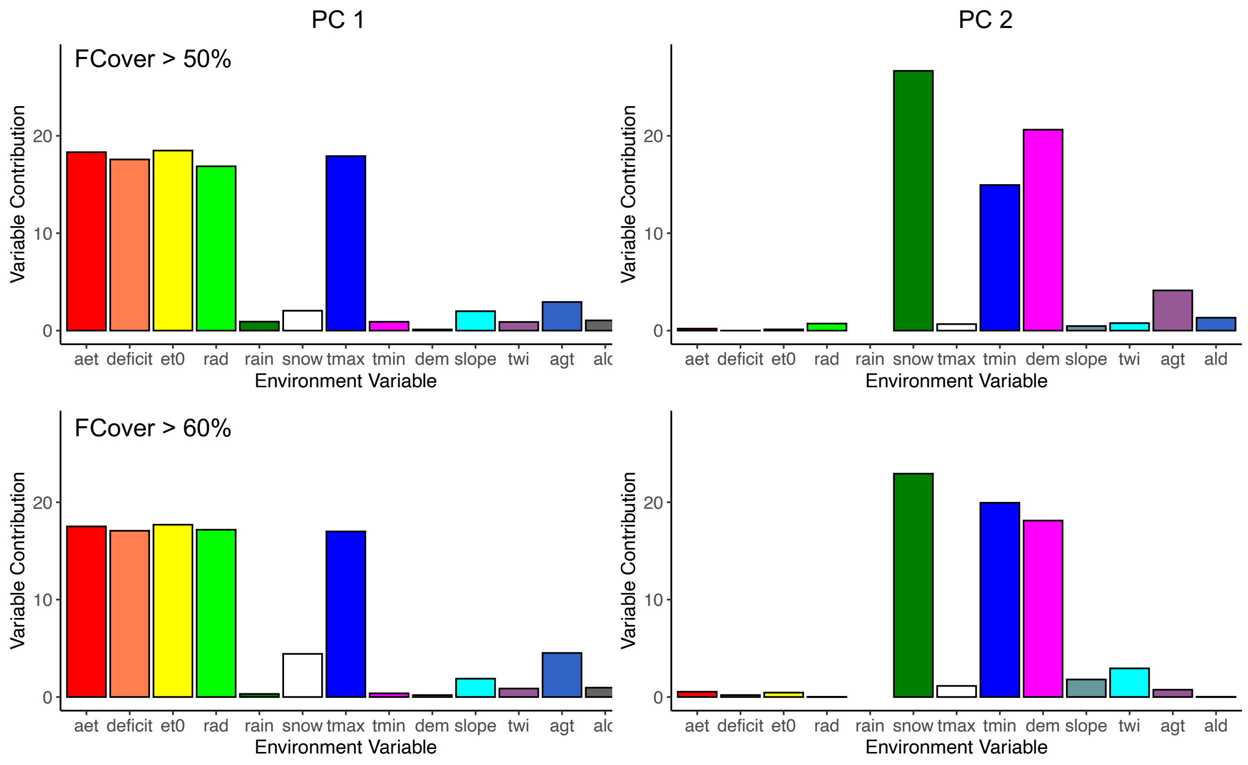

Fig. S8: Differences in environmental drivers for large *Alnus* and *Salix* communities with fCover >50%.

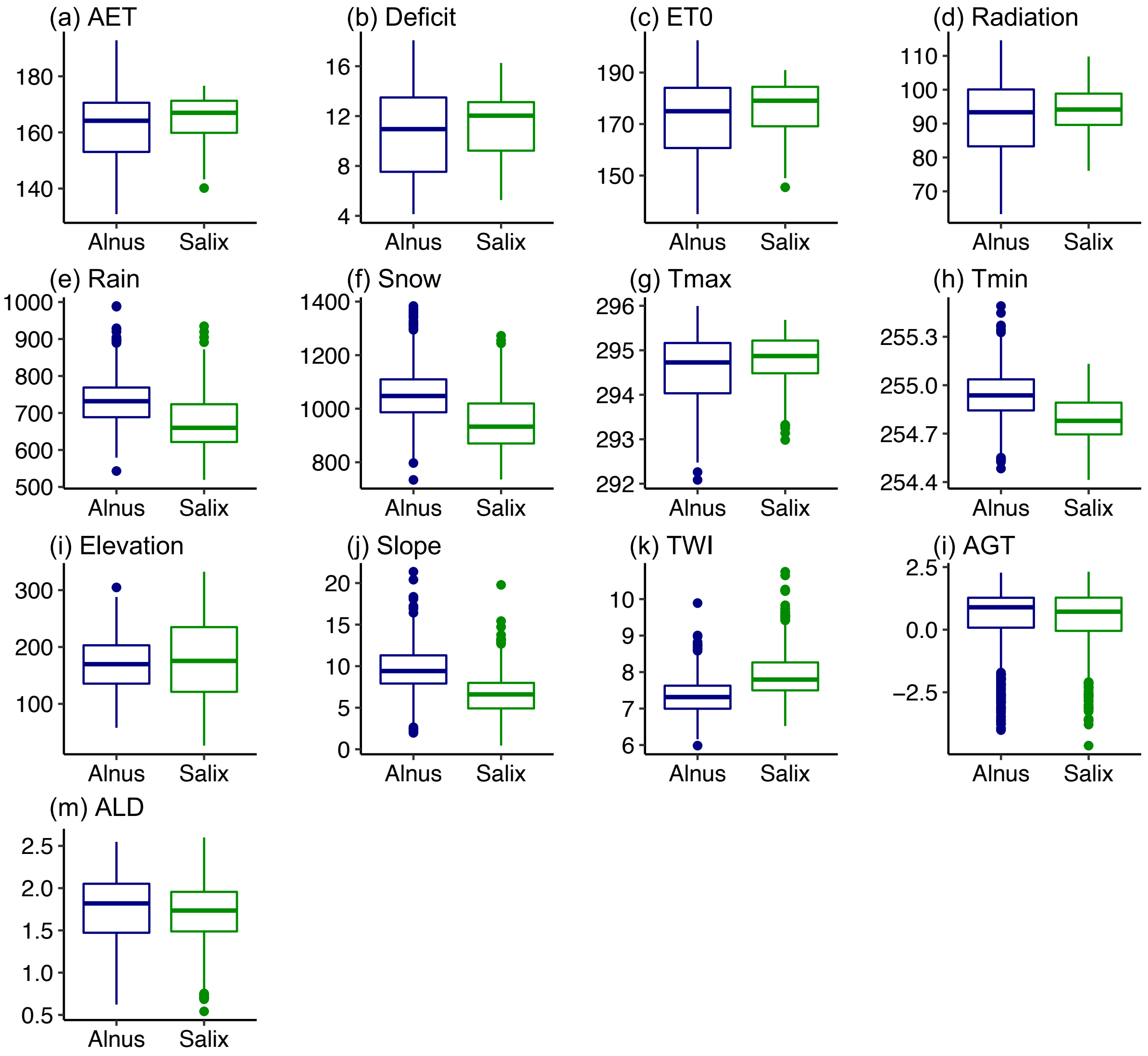

Fig. S9: Variation of stomatal slope (g1) across soil moisture gradient and key shrub community types.

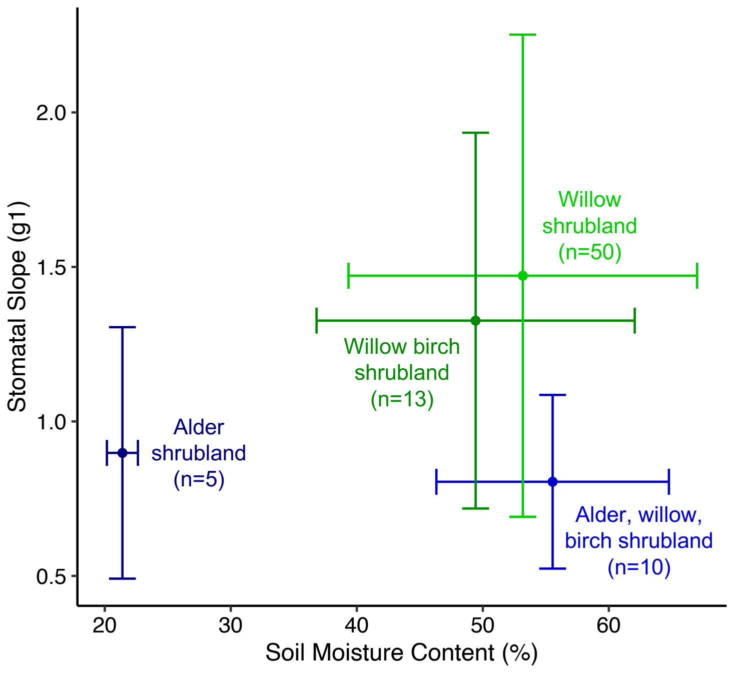

Fig. S10: Per-variable limiting factor models (i.e., maximum fCover response to environmental drivers) for *Alnus*. Colors in the plots represent point density.

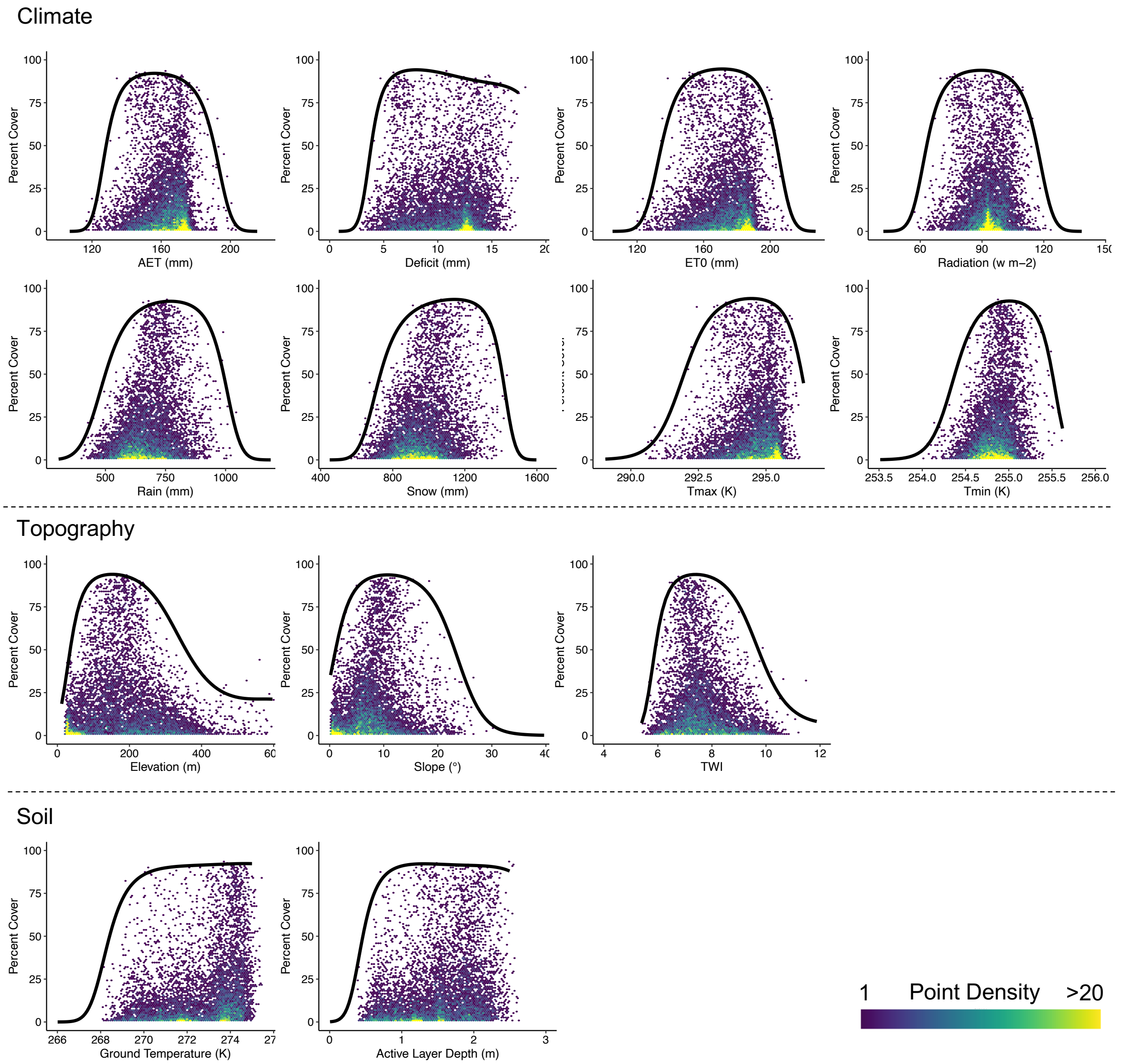

Fig. S11: Per-variable limiting factor models (i.e., maximum fCover response to environmental drivers) for *Salix*. Colors in the plots represent point density.

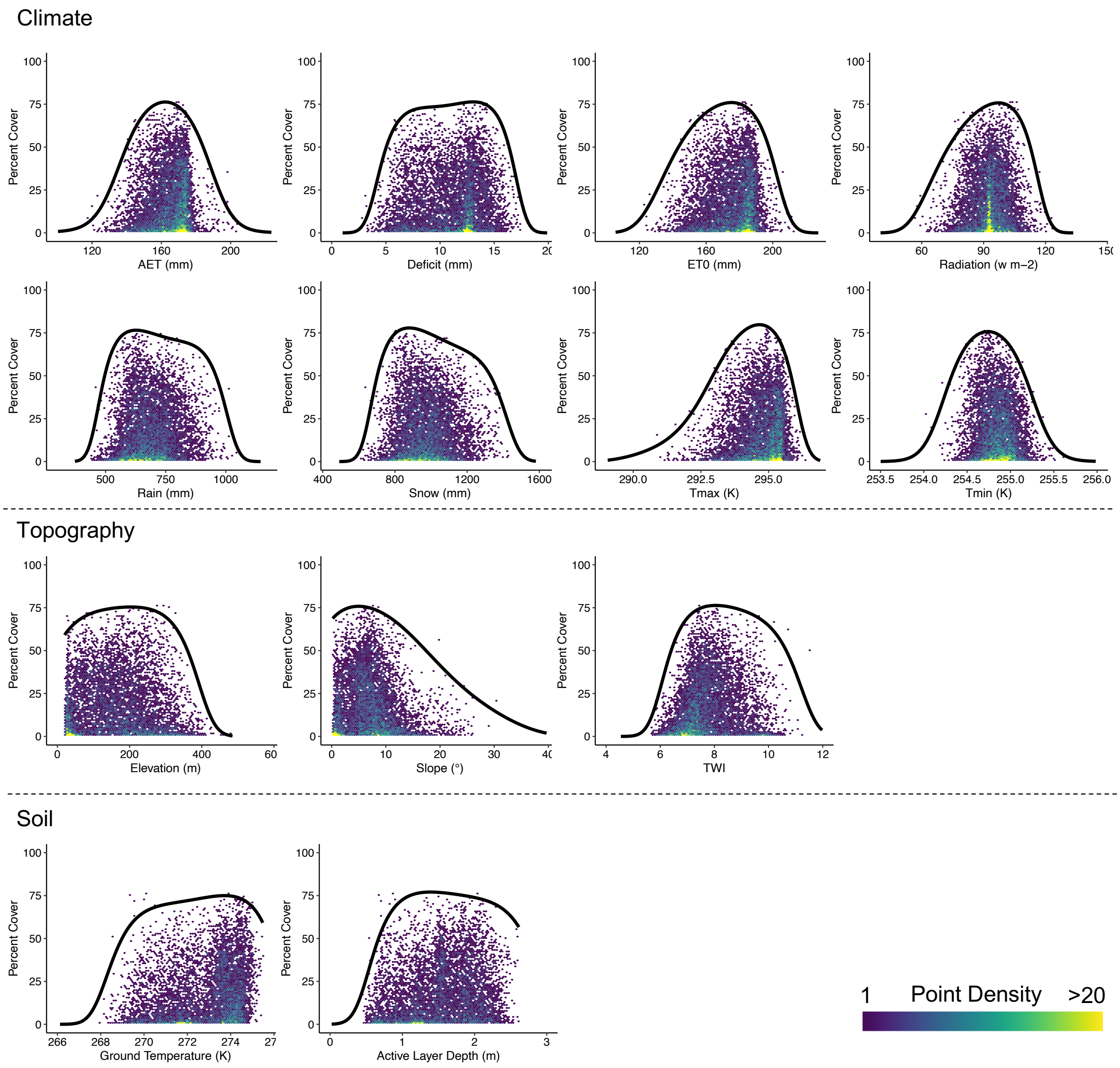

Fig. S12: Histogram distribution of *Alnus* and *Salix* environmental limiting factors (ELFs) across the landscape.

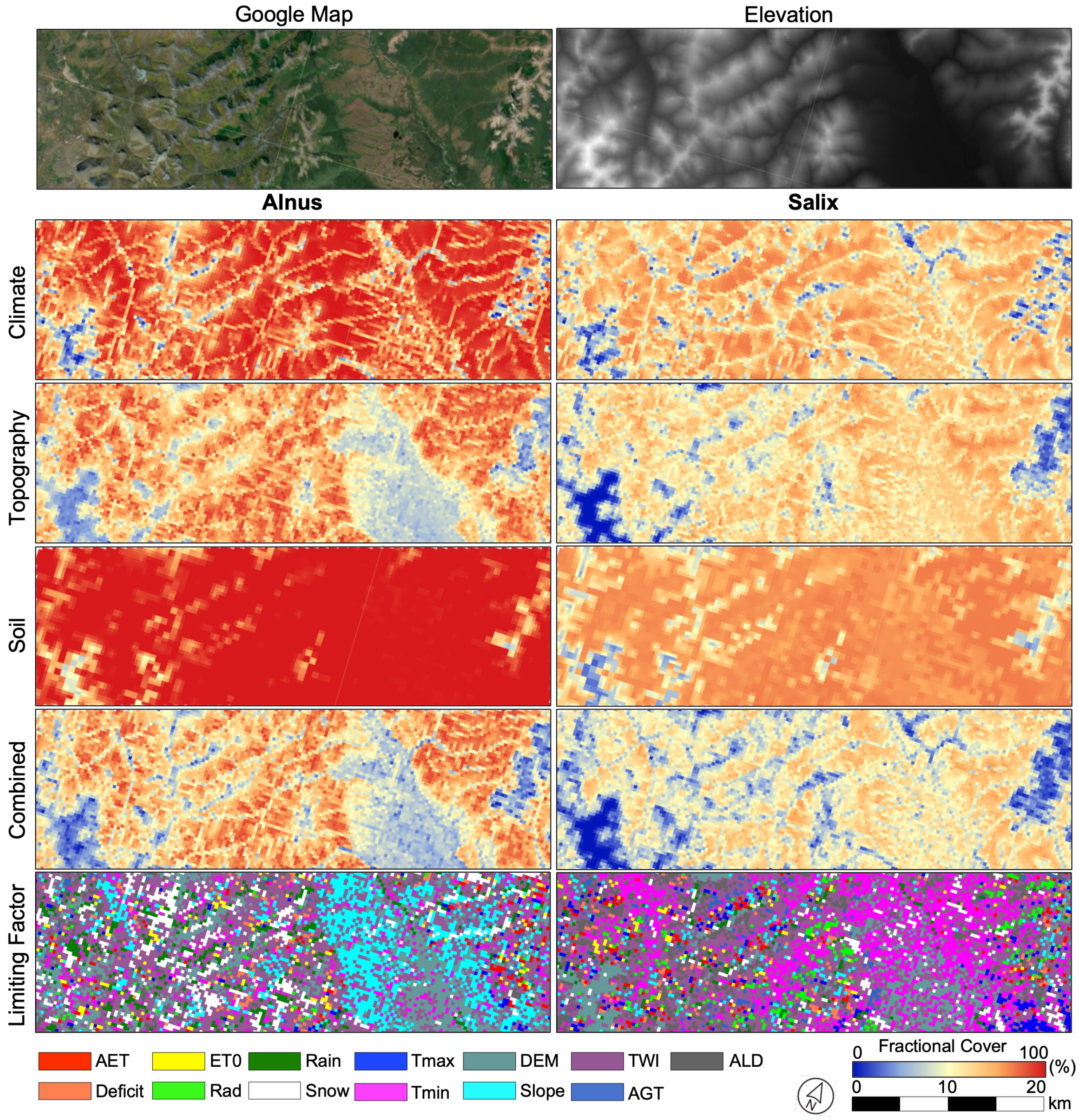

Fig. S13: ELFs of Alnus/Salix at regions that have low and high potential fCover, respectively.

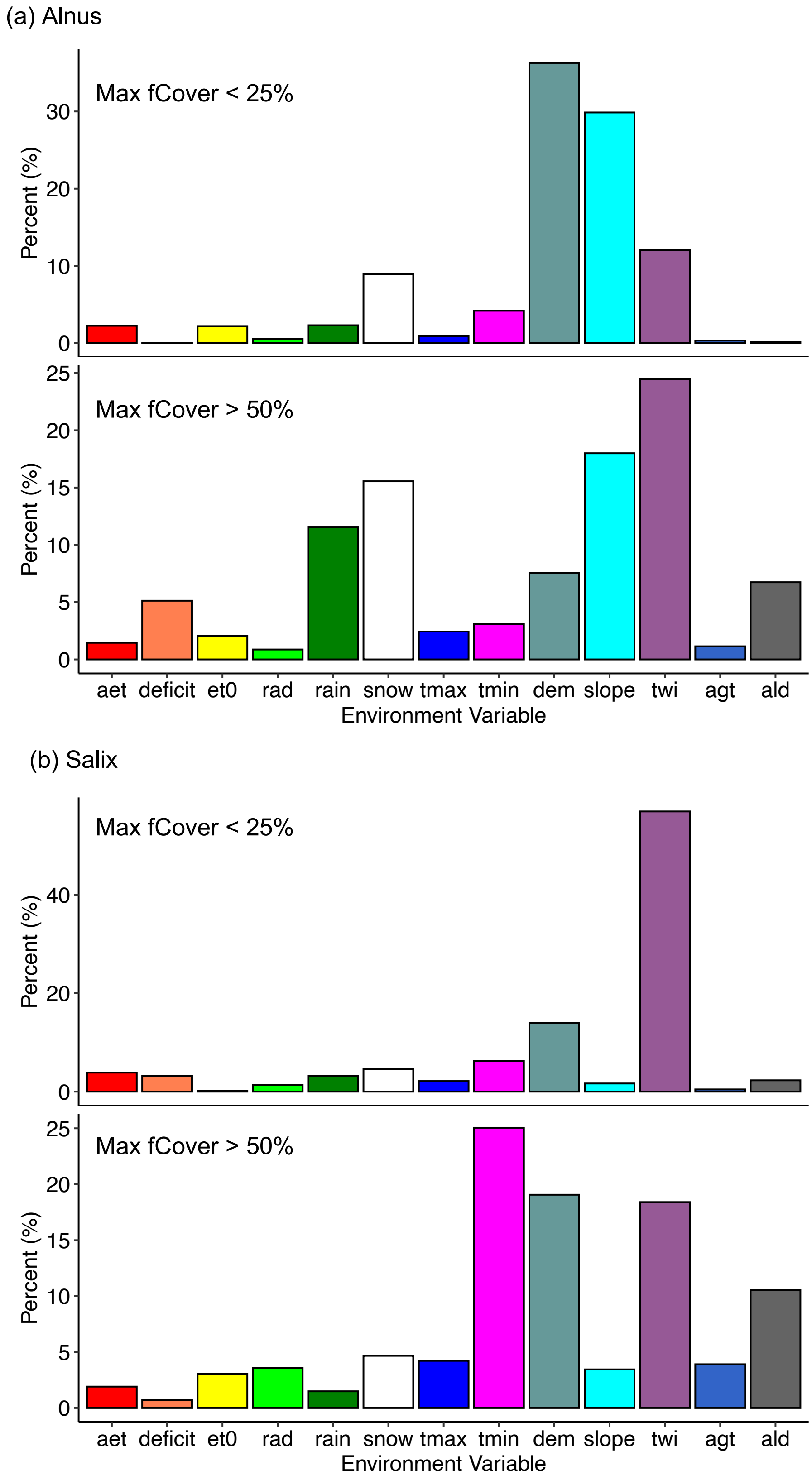

Fig. S14: Percent variance explained by different combinations of variables from a step-wise RF modeling analysis. In brief, topographic, soil, and climate variables were gradually added into the RF (based on the importance observed in Fig. 5). The variance for soil is additional variance in *Alnus* and *Salix* fCover explained beyond topographic variables. Similarly, the variance for climate is additional variance in *Alnus* and *Salix* fCover explain beyond the combination of topography and soil. This analysis confirmed the important contribution of topographic variables for determining the distribution of *Alnus* and *Salix* fCover.

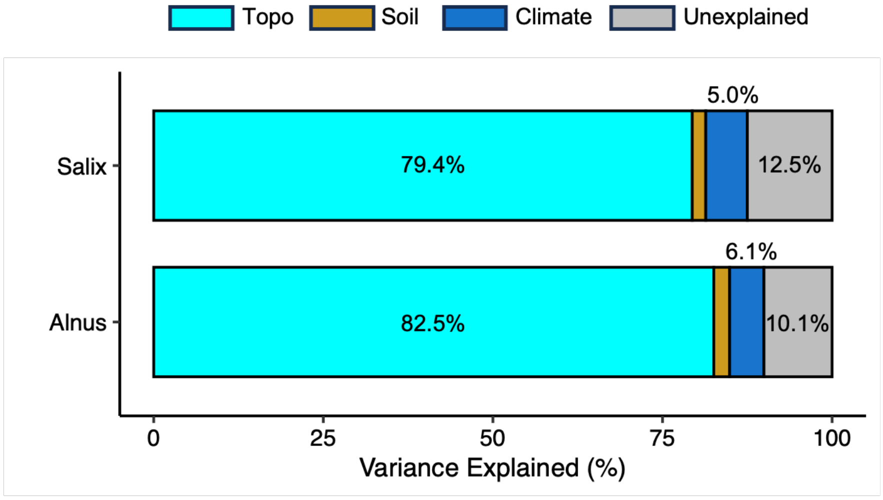

Table S1: *Alnus* and *Salix* mapping accuracy and overall classification accuracy from UAS imagery

	Alnus		Salix		All classes	
	Producer's accuracy	User's accuracy	Producer's accuracy	User's accuracy	Overall accuracy	Kappa coefficient
Flight a	88.34%	91.25%	92.46%	90.28%	89.28%	0.83
Flight b	90.12%	88.94%	93.71%	89.37%	91.92%	0.87
Flight c	89.27%	89.86%	95.83%	87.91%	88.63%	0.83
Flight d	91.25%	82.45%	95.75%	92.39	90.24%	0.86
