# Supplementary material for: Topography and functional traits shape the distribution of key shrub plant functional types in low-Arctic tundra
Source: Front Plant Sci. 2026 Jan 7;16:1724838. doi: 10.3389/fpls.2025.1724838 (PMC12819713; doi:10.3389/fpls.2025.1724838)
Supplement: Supplementary file 2 [file DataSheet2.pdf]

Journal: Frontiers in Plant Science

Title: Topography and Functional Traits Control the Distribution of Key Shrub Plant Functional Types in Low-Arctic Tundra

Author List: Daryl Yang[1,2,3*], Wouter Hantson[4,5], Kenneth J. Davidson[2,3,6], Julien Lamour[7], Bailey D. Morrison[8], Verity G. Salmon[1], Tianqi Zhang[1], Kim S. Ely[2,9], Charles E. Miller[10], Daniel J. Hayes[4], Stephen Baines[3], Alistair Rogers[2,9], Shawn P. Serbin[2,3,11]

Author Affiliation:

1. Environmental Sciences Division and Climate Change Science Institute, Oak Ridge National Laboratory, Oak Ridge, TN, USA
2. Environmental and Climate Sciences Department, Brookhaven National Laboratory, Upton, NY, USA
3. Department of Ecology and Evolution, Stony Brook University, Stony Brook, NY, USA
4. School of Forest Resources, University of Maine, Orono, ME, USA
5. WSL Institute for Snow and Avalanche Research SLF, Alpine Environment and Natural Hazards, Davos Dorf, Switzerland
6. American Forests, Washington DC, USA
7. Centre de Recherche sur la Biodiversité et l'Environnement (CRBE), Université de Toulouse, CNRS, IRD, Toulouse INP, Université Toulouse 3 – Paul Sabatier (UT3), Toulouse, France
8. Department of Civil and Environmental Engineering, University of California, Merced, Merced, CA, USA
9. Climate and Ecosystem Sciences Division, Berkeley National Laboratory, Berkeley, CA, USA
10. Jet Propulsion Laboratory, California Institute of Technology, Pasadena, CA, USA
11. Biospheric Sciences Laboratory (Code 618), NASA Goddard Space Flight Center, Greenbelt, MD, USA

***Corresponding author:** Daryl Yang (email: yangd@ornl.gov)

Contents of this file:

Support Materials

Introduction

This document contains details on leaf functional traits measurements to support results on trait differences between *Alnus spp*. and *Salix spp*. in "*Topography and Functional Traits Shape the Distribution of Key Shrub Plant Functional Types in Low-Arctic Tundra*" submitted to *Frontiers in Plant Science*. These details include field collection and laboratory processing, as well as trait calculation, for leaf biochemical, morphological, and physiological traits. Methods related to trait calculation and the resulting datasets are also included in References.

Support Materials

Leaf biochemical and morphological trait measurements:

Leaf samples used for quantifying *Alnus* and *Salix* biochemical and morphological traits were collected during the peak growing seasons of 2016 to 2019 at the Teller and Kougarok field sites (Fig. 1) established by the Next Generation Ecosystem Experiment Arctic (NGEE Arctic). Because the sites are a significant distance away from where we could lodge (45 minutes to Teller and 2 hours to Kougarok), foliar samples were stored in a cool, dark, and humid environment from the time they were removed from the plant to the time they were processed. This time did not exceed 4 hours. Once we returned to our lodge, we began processing samples immediately. First, using a metal punch of a known area, we extracted multiple leaf discs for each sample. The selection of the leaf punch depended on the size of the leaf being harvested, ranging from 2 cm^2 to 5 cm^2. Discs were taken from across the area of the leaf in order to account for the variation within a leaf. The fresh weight of the sampled discs was weighted using a high-accuracy, portable Fisher Science Education™ portable balance scale (Fisher Scientific Inc.). After that, we placed the samples in a laboratory oven (provided by University of Alaska, Fairbanks) for drying before transporting them back to Brookhaven National Laboratory (BNL) for further analysis. For biochemical and morphological trait analysis, we re-dried the leaf samples in a laboratory oven at BNL at 70 °C for at least 24 hours. Leaf dry mass was then determined using a top pan balance (AND ER-60A; A&D Engineering, San Jose, CA USA), which along with fresh mass and area allowed us to calculate leaf mass per area (LMA) and leaf water content (LWC).

Subsequently, dried leaves were ground to a powder using a ball mill, then samples for analysis were weighed into tin capsules on a Mettler Toledo AX26 DeltaRange mass balance (Mettler Toledo, Columbus, OH USA). Foliar elemental carbon and nitrogen content was quantified using a Perkin Elmer 2400 Series II CHNS/O Elemental Analyzer, used according to the manufacturer's instructions (Perkin Elmer, Waltham, MA USA). The resulted trait data are available at Serbin et al. (2023a, 2023b, 2023c) and are summarized in Table 2.

Leaf physiological trait measurement:

Leaf physiological traits were estimated from gas exchange measurements collected during the peak growing seasons of 2019, 2022, and 2023. To estimate V_{cmax} and J_{max}, we collected CO_2 response curves (AC_i curves) using four LI-6400XT Portable Photosynthesis Systems, each equipped with a 6400-02B LED light source set to a color spectrum of 90% red, 10% blue. Prior to the measurement campaign, instruments were zeroed using a common nitrogen standard. Before conducting AC_i curves, preliminary light response

curves were performed and examined for each species, to determine saturating irradiance (1800 µmol photons m^{-2} s^{-1} for all species) which would be used for all subsequent curves. During each AC_i curve, T_{leaf} was held close to ambient conditions. Following a minimum of 40 minutes of stabilization to chamber conditions, CO_2 was decreased by a number of set points from 400 micromol mol^{-1}, followed by an incremental increase to 1800 micromol mol-1, typically following the sequence 400, 300, 225, 150, 100, 75, 50, 400, 400, 400, 475, 575, 675, 800, 1000, 1400 and 1800 micromol mol-1. After adjusting each new CO_2 level, data were logged as soon as CO_2 and photosynthetic rates were stable after a minimum wait time of 60 seconds. CO_2 response curves were followed by dark respiration measurements on the same leaf after a dark adaptation time of at least 20 minutes. Once stability was reached, the analyzers were matched, and data was logged at 5-second intervals for 5 minutes. Using these measurements, we estimated V_{cmax} and J_{max} using the Farquhar-von Caemmerer-Berry (FvCB) model (Farquhar et al., 1980). The estimated V_{cmax} and J_{max} were then scaled to 25 °C, following Rogers et al., (2017). The resulted V_{cmax} and J_{max} data are available at Rogers et al. (2024) and are summarized in Table 2.

To estimate stomatal parameters (g_1 and g_0, we collected stomatal response curves using five LI-6800 Portable Photosynthesis Systems each equipped with a 6800-01A fluorometer light source. During each response curve, T_{leaf} and VPD_{leaf} were held at a fixed value close to ambient conditions, and chamber CO_2 was fixed at 400 mol mol-1. When steady-state gas exchange had been achieved (A and g_s were stable over a 5–10 min period), a log was recorded, with this process repeated across a range of irradiance levels (1500, 500, 200, 75 and 0 µmol m^{-2} s^{-1}). To estimate g_1 and $g_0$we used the unified stomatal optimization (USO) model (Medlyn et al., 2011, Eq. 1)

$$g_s = g_0 + 1.6(1 + \frac{g_1}{\sqrt{VPD_s}})\frac{A_n}{CO2_s} \quad \text{(Eq. 1)}$$

where g_0 (mol m^{-2} s^{-1}) represents the expected stomatal conductance when net photosynthesis is zero, D_s is the leaf to air vapor pressure deficit (kPa), and C_s is the CO_2 concentration at the leaf surface (mol mol−1). The result g0 and g1 data is available at Davidson & Serbin et al. (2024) and Ely et al. (2024). and are summarized in Table 2.

References:

Davidson K; Serbin S (2024): Stomatal response of three Arctic plant species, Seward Peninsula, Alaska, 2022. Next-Generation Ecosystem Experiments (NGEE) Arctic, ESS-DIVE repository. Dataset. doi:10.5440/1975097

Ely K; Yang D; Anderson J; Serbin S; Rogers A (2024): Plant physiology, shrub size, thaw depth and soil water content, Seward Peninsula, Alaska, 2023. Next-Generation Ecosystem Experiments (NGEE) Arctic, ESS-DIVE repository. Dataset. doi:10.15485/2341585

Farquhar G. D., von Caemmerer S. & Berry J. A. (1980). A biochemical model of photosynthetic CO2 assimilation in leaves of C 3 species. *Planta*, *149*(1), 78–90.

Medlyn, B. E., Duursma, R. A., Eamus, D., Ellsworth, D. S., Prentice, I. C., Barton, C. V. M., Crous, K. Y., De Angelis, P., Freeman, M., & Wingate, L. (2011). Reconciling the optimal and empirical approaches to modelling stomatal conductance. *Global Change Biology*, *17*(6), 2134–2144.

Rogers, A., Serbin, S. P., Ely, K. S., Sloan, V. L., & Wullschleger, S. D. (2017). Terrestrial biosphere models underestimate photosynthetic capacity and CO2 assimilation in the Arctic. New Phytologist, 216(4), 1090–1103.

Rogers A; Ely K; Davidson K (2024): Plant Physiology, Alder and Willow Species, Seward Peninsula, Alaska, 2019. Next-Generation Ecosystem Experiments (NGEE) Arctic, ESS-DIVE repository. Dataset. doi:10.5440/1696801

Serbin S; Meng R; McMahon A; Yang D; Ely K; Rogers A (2023a): Leaf Nitrogen, Leaf Mass Area, Leaf Water Content, Seward Peninsula, Alaska, 2017. Next-Generation Ecosystem Experiments (NGEE) Arctic, ESS-DIVE repository. Dataset. doi:10.5440/1783188

Serbin S; Yang D; Ely K (2023b): Leaf Nitrogen and Carbon Content, and Leaf Mass Per Area, Kougarok Road, Seward Peninsula, Alaska, 2018. Next-Generation Ecosystem Experiments (NGEE) Arctic, ESS-DIVE repository. Dataset. doi:10.5440/1631419

Serbin S; Rogers A; Yang D; Davidson K; Ely K (2023c): Leaf structural and chemical traits, and vegetation temperature and height, Seward Peninsula, Alaska, 2019. Next-Generation Ecosystem Experiments (NGEE) Arctic, ESS-DIVE repository. Dataset. doi:10.5440/1783192
